# Restoring pre-osteoarthritis cartilage matrix integrity via Col9 mRNA halts osteoarthritis progression

**DOI:** 10.1126/sciadv.aef9739

**Published:** 2026-09-02

**Authors:** Jun Zhou, Jingrui Hu, Erica Yada, Jingxiao Zhong, Yuki Terai, C. Peter Winlove, Junning Chen, Yo Shibata, Keiji Itaka

**Affiliations:** ^1^ Clinical Biotechnology Team, Center for Infectious Disease Education and Research (CiDER), The University of Osaka, Suita, Osaka 565-0871, Japan.; ^2^ Department of Biomaterials and Engineering, Showa Medical University Graduate School of Dentistry, Shinagawa-ku, Tokyo 142-8555, Japan.; ^3^ Institute of Integrated Research, Institute of Science Tokyo, Bunkyo-ku, Tokyo 113-8656, Japan.; ^4^ Biomedical Engineering, Department of Engineering, Faculty of Environment, Science and Economy, University of Exeter, Exeter EX4 4RN, UK.; ^5^ School of Aerospace, Mechanical and Mechatronic Engineering, The University of Sydney, Sydney, NSW 2006, Australia.; ^6^ Biophysics, Faculty of Environment, Science and Economy, University of Exeter, Exeter, UK.

## Abstract

Osteoarthritis (OA) is a prevalent degenerative joint disease characterized by the progressive breakdown of cartilage. Here, we sought to define a pre-osteoarthritis (pre-OA) state using nanoindentation-based mechanical testing and evaluation of collagen organization in cartilage matrix. The reductions in elastic modulus and impaired viscoelasticity preceded any overt histological degeneration. This mechanical dysfunction was primarily driven by early disorganization of type II collagen, occurring before substantial depletion of cartilage components. We subsequently identified type IX collagen (Col IX), which played a critical role at the pre-OA stage. Intra-articular administration of *Col9* mRNAs delivered by polymer-based nanomicelle restored pre-OA cartilage to a healthy, native-like collagen network architecture and preserved cartilage mechanical integrity, ultimately halting OA progression in the rat model. These findings identify viscoelastic dysfunction as an early biomechanical hallmark of pre-OA cartilage and establish *Col9* mRNA as a potential anti-OA drug to prevent its onset. Together, these results provide a translatable framework in which quantitative mechanical-ultrastructural metrics guide ultra-early, presymptomatic intervention for degenerative diseases.

## INTRODUCTION

Osteoarthritis (OA) is a progressive disorder of the entire joint (*1*, *2*), characterized by the gradual erosion of the cartilage architecture and loss of its biomechanical competence (*3*). The extracellular matrix (ECM), which comprises up to 90% of cartilage volume, is primarily composed of densely packed fibrils of type II collagen (Col II) embedded in a highly hydrated proteoglycan (PG) aggrecan gel (*4*, *5*). Chondrocytes, whose principal function is to maintain ECM homeostasis, exhibit low metabolic activity, and the solute impermeability and avascular nature of cartilage severely limit its ability to repair following degeneration. By the time OA becomes clinically symptomatic and radiographically detectable, irreversible cartilage damage has often already occurred, leaving few therapeutic options beyond costly, late-stage surgical interventions (*6*–*8*). These challenges underscore the urgent need to identify the presymptomatic phase of OA, thereby facilitating the development of disease-modifying OA drugs capable of preserving cartilage structure, mechanical integrity, and function.

Recent advances in biomechanical testing and analytical techniques have improved the sensitivity of detecting the earliest functional changes in tissues (*9*). Indentation-type mechanical probes have demonstrated high efficacy in characterizing the viscoelastic properties of cartilage (*10*–*13*). Spatially resolved creep compliance measurements can quantify key mechanical parameters that capture both tissue elasticity and viscosity, offering mechanistic insights into the dynamic interplay among matrix components such as collagen fibrils, macromolecules, and interstitial fluid (*14*). In addition, polarization-resolved second harmonic generation (pSHG) microscopy has emerged as a powerful, label-free imaging technique for assessing collagen intrafibrillar organization, including its principal orientation and molecular packing (*15*–*17*).

Herein, we investigated structural and mechanical alterations in the cartilage ECM during the pre-disease phase using an instability-induced OA model. Cartilage dysfunction begins with an early disruption of the collagen network, occurring before overt matrix degradation. To elucidate the molecular mechanisms underlying this disruption, transcriptomic analysis revealed a notable down-regulation of *Col9* genes. The encoded protein, type IX collagen (Col IX), covalently cross-links Col II fibrils and anchors them within the PG network, providing structural stability and mechanical resilience (*18*, *19*). Although genetic alterations in *Col9* genes have been linked to early OA (*20*), its therapeutic potential remains unclear.

On the basis of these findings, we hypothesized that restoring *Col9* expression at OA onset could prevent cartilage degeneration by preserving cartilage matrix integrity. Among emerging therapeutic modalities, mRNA technologies have shown great promise for targeted biomedical interventions (*21*). mRNA therapy can directly modulate intracellular signaling and maintain ECM homeostasis through the rapid translation of therapeutic proteins, highlighting its potential as a newly identified treatment strategy for OA (*22*). Therefore, in this study, we developed an mRNA therapy encoding *Col9a1* and *Col9a2* and proposed that the resulting exogenous Col IX protein could maintain the integrity of the cartilage matrix during the pre-OA stage.

## RESULTS

### Morphological tracking of early OA progression

**Fig. 1.**
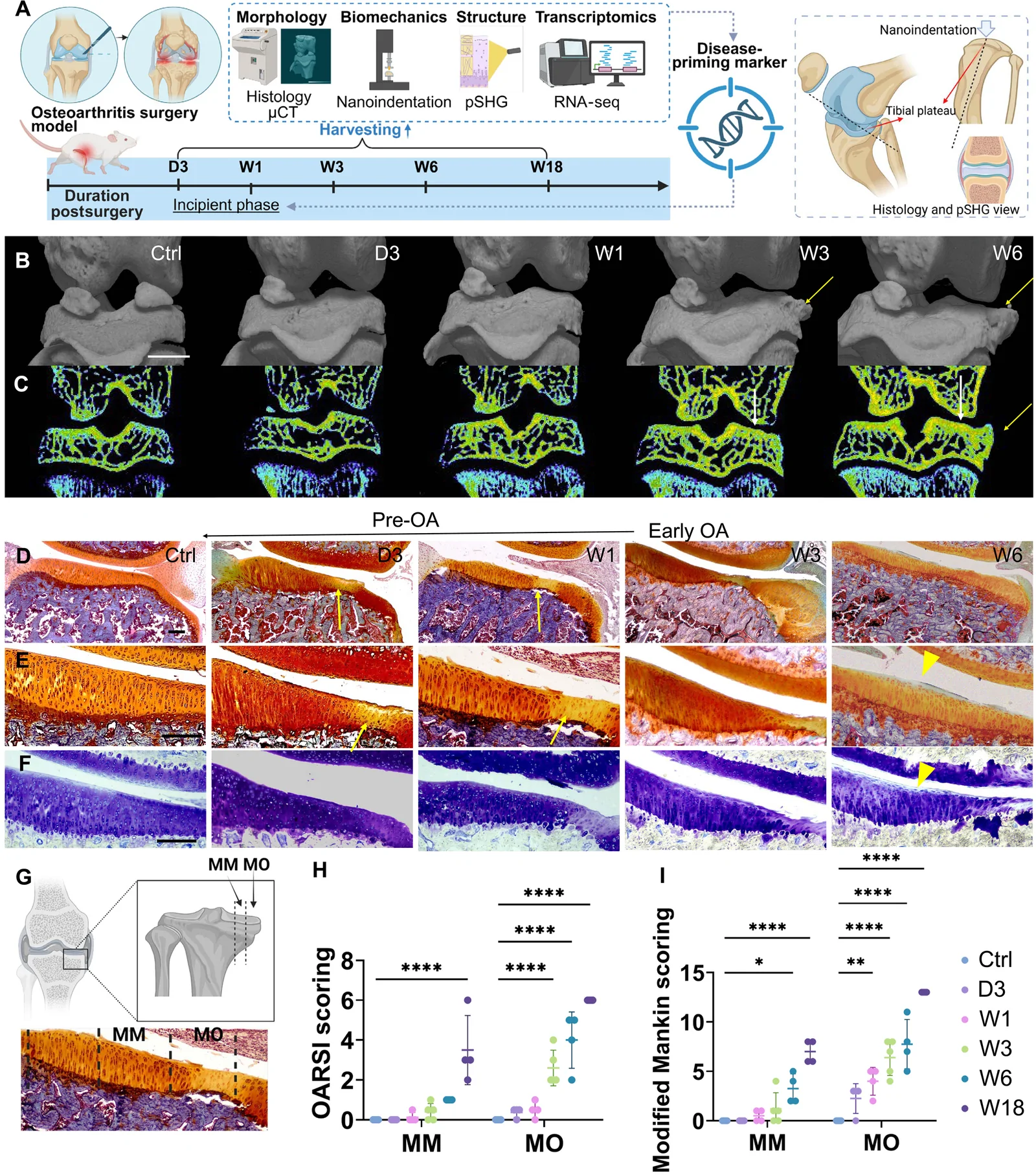
Representative μCT and histological analyses reveal predominant cartilage lesions in the MO region during the onset of OA. (**A**) Schematic of the study design and test views, illustrating the experimental workflow including histology, microcomputed tomography (μCT), nanoindentation, pSHG, and RNA sequencing (RNA-seq). OA was induced in rats via medial meniscectomy (MMx), with samples collected at multiple postsurgery time points. (**B**) Representative μCT reconstructions of the femoral condyle and tibial plateau (scale bar, 2 mm). (**C**) Coronal μCT sections highlighting osteophyte formation (yellow arrows) and joint space narrowing (JSN; white arrows). (**D**) Safranin O/Fast Green staining of medial tibial cartilage at various OA stages. (**E**) Magnified Safranin O/Fast Green–stained sections showing early PG loss (yellow arrows) in the medial outer (MO) region and surface staining loss in medial middle (MM) cartilage by W6 (yellow triangle). (**F**) Toluidine blue staining showing progressive matrix depletion over time (scale bars, 200 μm). (**G**) Representative image at W1 postsurgery demonstrating site-specific changes in the PG content, with no changes in MM cartilage but overt PG loss in MO. (**H** and **I**) OARSI and modified Mankin scores (means ± s.d.; *n* ≥ 4) for site-specific OA progression. Statistical significance was assessed by two-way analysis of variance (ANOVA) followed by Tukey’s multiple comparisons test (*P < 0.05, **P < 0.01, and ****P < 0.0001). (A and G) Created in BioRender. Zhou, J. (2026); https://BioRender.com/5junwc2. D3, 3 days; W1, 1 week; W3, 3 weeks; W6, 6 weeks; W18, 18 weeks.

To track OA progression, we performed microcomputed tomography (μCT) and histological analyses of rat knee joints at 3 days (D3), 1 week (W1), 3 weeks (W3), 6 weeks (W6), and 18 weeks (W18) following medial meniscectomy (MMx; Fig. 1A). Based on current diagnostic criteria for human OA, we evaluated and compared osteophyte formation and joint space narrowing (JSN) in our μCT images across the different experimental groups. Three-dimensional μCT reconstructions revealed the onset of osteophyte formation at W3, which progressed substantially by W6 (Fig. 1, B and C), and, by W18, the osteophytes were large and densely mineralized (fig. S1, A and B). Color-coded coronal sections demonstrated that the JSN in the medial tibial condyle first appeared at W3 and was consistently observed at W6 and W18 (fig. S1C). Based on the Kellgren-Lawrence grading system for human OA (*23*), W3 in the rat MMx OA model corresponds to grade 1 (early-stage) OA in humans, characterized by initial JSN and osteophytic lipping (fig. S1D).

Histopathological analysis using Safranin O/Fast Green and toluidine blue staining revealed distinct spatial patterns of PG depletion in cartilage. The medial outer (MO) region showed PG loss as early as D3, which became pronounced by W1, and hyaline cartilage was severely eroded by W18 (Fig. 1, D to F, and fig. S2). Conversely, cartilage in the medial middle (MM) region maintained its PG content until W6, when distinct surface depletion emerged (Fig. 1, E and F). These findings demonstrate region-dependent trajectories of OA progression in the joint-instability animal OA model (Fig. 1G). Osteoarthritis Research Society International (OARSI) (*24*) and modified Mankin scores further confirmed the site-specific progression of OA (Fig. 1, H and I). Although the MO cartilage showed significantly increased scores after W1 (*P* < 0.01), the MM cartilage displayed no significant changes until W6 (modified Mankin, *P* < 0.05) or W18 (OARSI, *P* < 0.0001). This disparity resulted from greater mechanical overloading in the MO region versus relative underloading in the MM region (*24*), with the latter more accurately recapitulating the progressive degenerative features of human OA. Based on these morphological findings, we defined D3 and W1 as the pre-OA stage and selected the MM region as the primary region of interest for investigating pre-OA phenotypes.

### Viscoelastic dysfunction precedes morphology

**Fig. 2.**
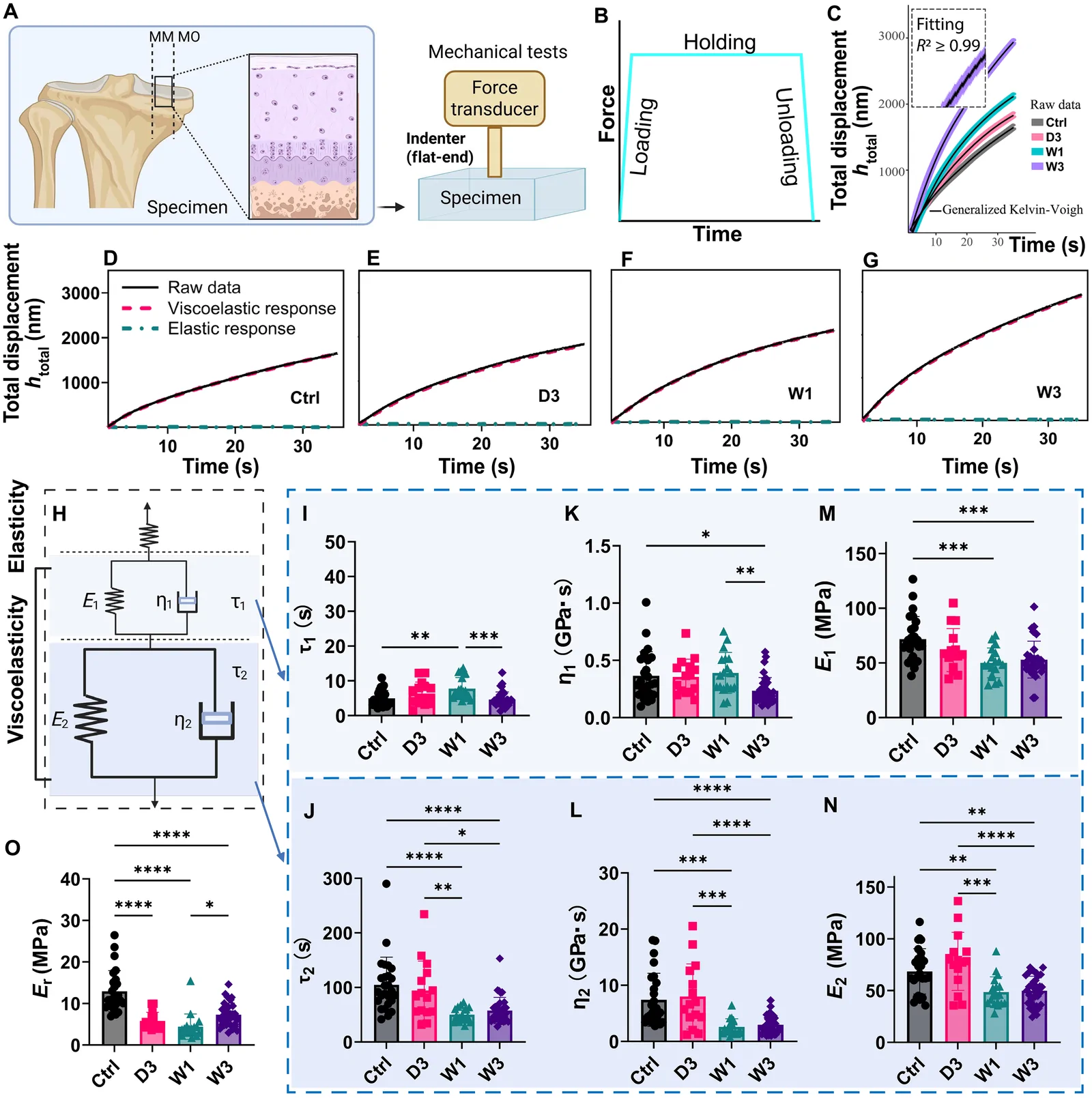
Time-dependent viscoelastic behavior of cartilage in early OA. (**A**) Schematic of the nanoindentation setup using a 10-μm flat-end probe. (**B**) Creep loading protocol comprising loading, a 35-s hold, and unloading. (**C**) Representative holding curves of MM cartilage in early OA. Black lines indicate curve fitting using the generalized Kelvin-Voigt (KV) model [coefficient of determination (*R*^2^) ≥ 0.99]. (**D** to **G**) Representative viscoelastic and elastic responses on MM cartilage during the hold phase at different OA stages. (**H**) Schematic diagram of the KV model, highlighting the second main body of the viscoelastic phase. (**I** to **N**) Mechanical parameters of the viscoelastic phase, including retardation times τ_1_ (I) and τ_2_ (J), viscosity coefficients η_1_ (K) and η_2_ (L), and elastic moduli *E*_1_ (M) and *E*_2_ (N). (**O**) Mechanical parameters of the elastic phase, represented by the *E*_r_ value. Data are presented as means ± s.d.; *n* ≥ 3 animals per group; *n* ≥ 4 nanoindentation tests per sample; in total, *n* > 12 repeats per group. Statistical significance was determined using one-way ANOVA with Tukey’s multiple comparisons test (*P < 0.05, **P < 0.01, ***P < 0.001, and ****P < 0.0001). (A and H) Created in BioRender. Zhou, J. (2026) https://BioRender.com/bszub18. D3, 3 days; W1, 1 week; W3, 3 weeks.

To characterize the mechanical alterations during OA progression, we performed nanoindentation testing on the MM region of cartilage samples (Fig. 2A). The instantaneous elastic modulus (*E*_inst_) decreased significantly by D3 (*P* < 0.01), reaching its lowest value (3.6 ± 2.0 MPa) at W1, approximately one-third that of the control (Ctrl) (fig. S3). To elucidate the viscoelastic response of this gel-like, shock-absorbing matrix, a creep-loading protocol was used (Fig. 2B), and the resulting creep curves were fitted using the generalized Kelvin-Voigt (KV) model (*25*, *26*), with coefficient of determination (*R*^2^) ≥ 0.99 (Fig. 2C).

Decomposition of the total displacement revealed that viscoelastic contributions predominated over elastic responses (Fig. 2, D to G, red dashed lines), increasing progressively from D3 to W3. Quantitative analysis of viscoelastic parameters derived from the generalized KV model (Fig. 2H) revealed that the second constitutive component (τ_2_ *=* η_2_*/E*_2_) had a retardation time more than 20-fold greater than that of the first (τ_1_ = η_1_/*E*_1_), with mean values of 105 s (τ_2_; Fig. 2J) and 5 s (τ_1_; Fig. 2I), respectively in the Ctrl. This difference in retardation time primarily arose from the viscosity coefficients (η_1_ = 0.4 GPa·s and η_2_ = 7.4 GPa·s) as *E*_1_ and *E*_2_ were comparable (Fig. 2, K to N), suggesting that viscous behavior plays a major role in the viscoelastic response of cartilage as a biomaterial. By W1, τ_2_ decreased to 50.2 ± 12.8 s (Fig. 2J), driven by reductions in both η_2_ (Fig. 2L) and *E*_2_ (Fig. 2N), with the decline in η being more pronounced. The reduction in τ_2_, a key parameter of time-dependent viscoelasticity, indicated compromised viscoelasticity and, consequently, decreased shock absorption capacity. Consistent with the *E*_inst_ data (fig. S3), the time-independent reduced modulus (*E*_r_) in the MM region decreased to 4.4 ± 0.9 MPa at W1, approximately one-third that of the Ctrl (Fig. 2O). Consistent trends in both instantaneous modulus and reduced modulus confirmed temporal mechanical changes in the MM zone between intact and OA cartilage, excluding positional variability as a confound. These findings demonstrate that an early viscoelastic dysfunction in pre-OA cartilage precedes any observable morphological changes.

### Col II disorganization from W1

**Fig. 3.**
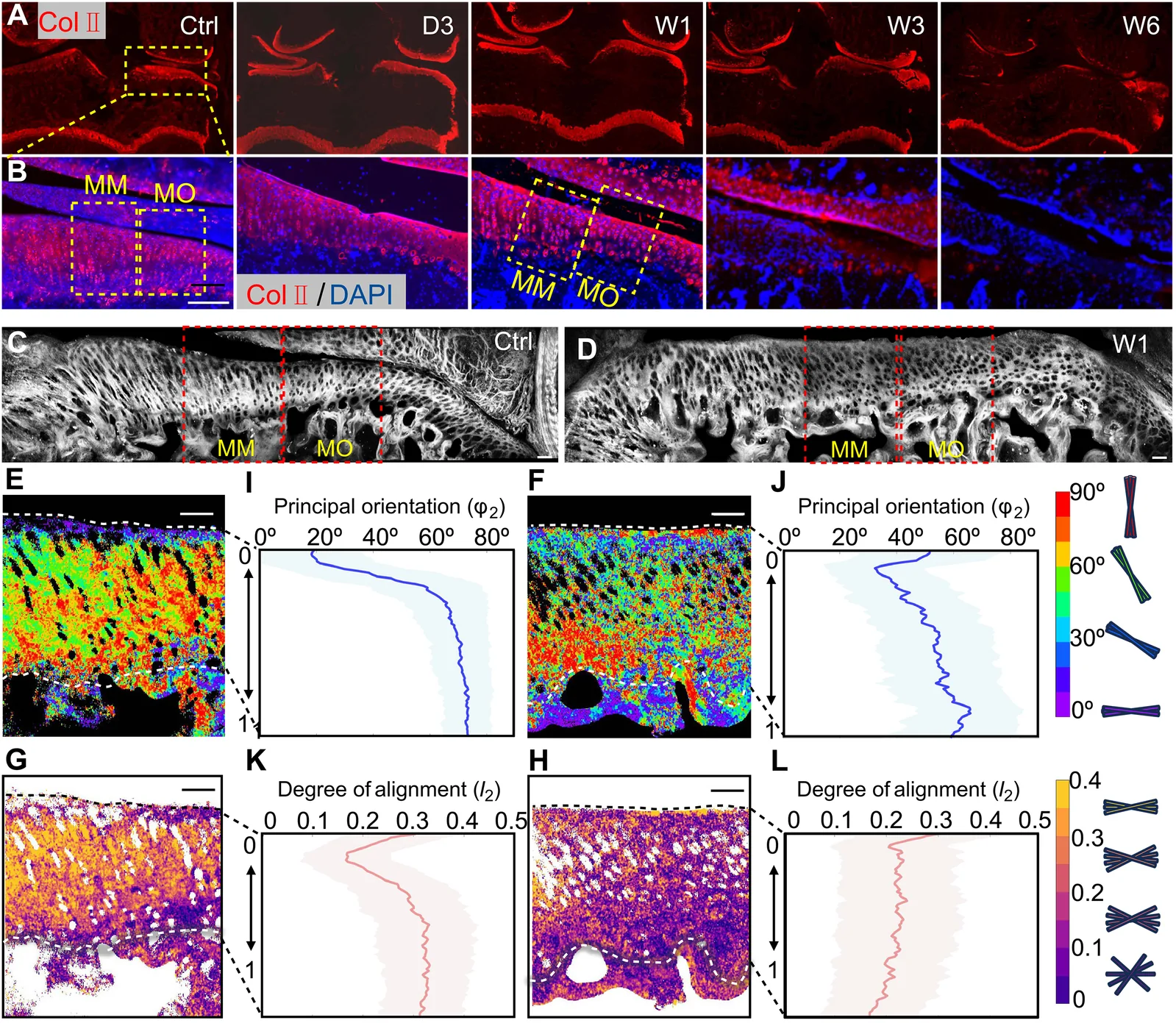
Ultrastructural changes in collagen fibril organization in the MM region of cartilage in intact (Ctrl) and W1 postsurgery cartilage. (**A**) Representative immunofluorescence staining for type II collagen (Col II) in cartilage sections at different stages of OA progression (intact to W6 postsurgery). (**B**) Magnified Col II and 4′,6-diamidino-2-phenylindole (DAPI) images, showing detailed Col II changes in the cartilage matrix during OA progression (scale bar, 200 μm). (**C** and **D**) Representative overview images of collagen in medial cartilage obtained by summing SHG signal across all polarization angles. (**E** and **F**) Pixel-based heat maps showing the principal orientation of collagen fibrils in MM regions, with representative examples from the Ctrl and W1 groups (*n* = 3). Colors closer to purple indicate fibrils more parallel to the cartilage surface, whereas colors closer to red indicate fibrils more perpendicular to the cartilage surface. (**G** and **H**) Heatmaps depicting the degree of alignment of collagen fibrils in MM regions, corresponding to those in (E) and (F). Brighter colors indicate higher fibril alignment (scale bars, 50 μm). (**I** and **J**) Depth-dependent line plots of (E) and (F), derived from masked cartilage regions with the subchondral bone plate excluded, showing the mean principal orientation of collagen fibrils (blue lines) within each row and their heterogeneity (light blue shading) across cartilage depth, normalized from articular surface to cement line. (**K** and **L**) Corresponding depth-dependent line plots for (G) and (H), showing the row mean values of collagen fibril alignment (red lines) and the heterogeneity within each row (light red shading), normalized from cartilage surface to cement line. D3, 3 days; W1, 1 week; W3, 3 weeks; W6, 6 weeks.

To determine whether mechanical failure arose from component loss or structural disruption in the matrix, we examined Col II content and collagen organization during OA progression. Immunostaining revealed that Col II content in the MM region remained largely preserved at W1, with marked reductions observed at W3 and W6 (Fig. 3, A and B).

pSHG microscopy was then applied to quantify ultrastructural changes in Col II intrafibrillar organization, specifically assessing the principal orientation and molecular packing throughout the tissue depth (Fig. 3, C to L). In intact cartilage (Ctrl), fibrils in the superficial zone were predominantly aligned parallel to the articular surface (Fig. 3, E and I), forming a smooth, well-organized layer. At W1, fibrils exhibited a pronounced upward tilt (Fig. 3, F and J), resulting in increased surface roughness and a reduced ability to resist compressive and shear forces (Fig. 3, F and J). In the deep zone, the normally vertical fibrils became flattened at W1, with orientation heterogeneity increasing throughout the tissue depth. By W3, zonal organization was completely lost (fig. S4A). Both the principal orientation (Fig. 3, F and J versus E and I) and molecular packing (Fig. 3, H and L versus G and K) revealed the loss of native gradients. Under control conditions within intact cartilage, the degree of order was lowest in the middle zone and increased toward the deep zone (Ctrl, ∼0.3); however, at W1, the fibrils exhibited loose packing (∼0.2) below the superficial zone.

Collectively, in pre-OA cartilage (W1, MM region), collagen network disorganization appeared before the obvious loss of matrix components. This structural disruption coincided with the reduction in τ_2_, suggesting that the early decline in time-dependent viscoelasticity is primarily driven by molecular-level architectural alterations rather than by the depletion of Col II or PG. These results identified a transitional stage of OA in which tissue integrity is determined by matrix organization rather than compositional content (fig. S4B).

### Transcriptomic changes from D3

**Fig. 4.**
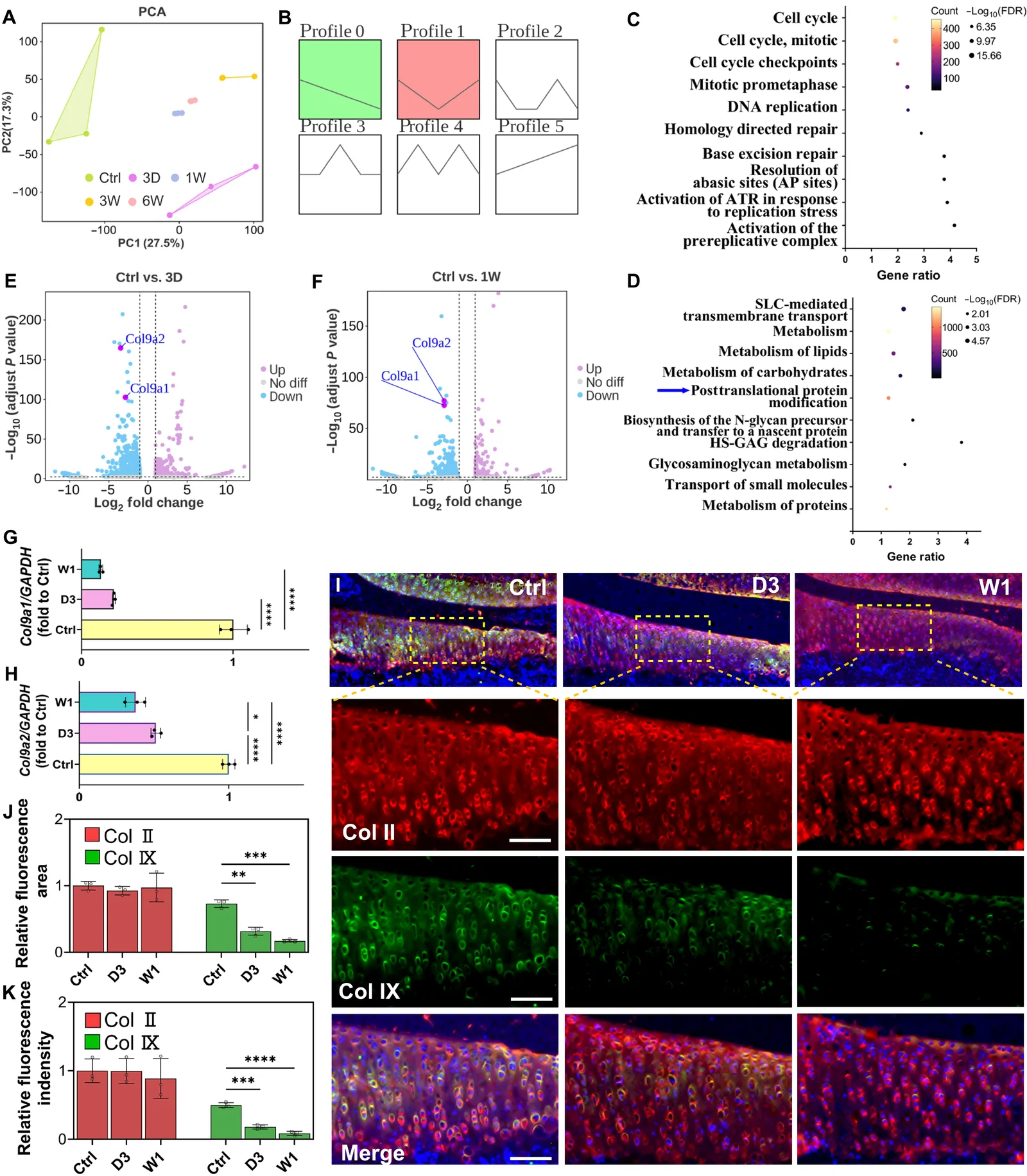
Col9 as an ECM integrity marker during the pre-OA phase. (**A**) Principal components analysis (PCA) of cartilage samples at different stages of OA, including intact cartilage (Ctrl); day 3 postinjury; and 1, 3, and 6 weeks postsurgery. (**B**) Short time-series expression miner (STEM) profiling. Profiles 0 and 1 are highlighted in color, representing significant profiles, ranked by *P* values. (**C** and **D**) Reactome pathway enrichment analysis for genes in profile 0 (C) and profile 1 (D). The most enriched pathways are shown with their respective gene counts and FDR values. (**E** and **F**) Volcano plots of differentially expressed genes (DEGs) at 3 days (**E**) and 1 week (**F**) relative to Ctrl, identifying genes with significantly altered expression, including *Col9a1* and *Col9a2*. (**G** and **H**) Reverse transcription quantitative polymerase chain reaction (RT-qPCR) validation of *Col9* gene expression from injured rat tibial cartilage at the pre-OA stage. Gene expression levels were calculated using *Gapdh* as a reference and normalized to those of Ctrl. (**I**) Representative immunofluorescence images of articular cartilage in the medial tibia stained for Col II (red), Col IX (green), and nuclei (DAPI; blue) in the Ctrl, D3, and W1 groups. Merged images show spatial colocalization of Col II and Col IX (scale bars, 100 μm). (**J** and **K**) Quantification of Col II and Col IX, including positive fluorescent area (J) and fluorescence intensity (K). Data are presented as means ± s.d. (*n* = 3). Statistical analysis was performed using one-way ANOVA (G and H) or two-way ANOVA (J and K) followed by Tukey’s multiple comparisons test (*P < 0.05, **P < 0.01, ***P < 0.001, and ****P < 0.0001). D3, 3 days; W1, 1 week; W3, 3 weeks; W6, 6 weeks.

Building on the observed structural and mechanical alterations in the pre-OA stage, we performed RNA sequencing (RNA-seq) to investigate chondrocyte-driven regulation of ECM architectural maintenance. Principal components analysis (PCA) showed distinct clustering by time point, indicating marked transcriptomic divergence by D3 (Fig. 4A). Short time-series expression miner (STEM) analysis identified two statistically significant temporal profiles (out of six; Fig. 4B). Profile 0 (5411 genes) displayed a continuous decline until W6 (fig. S5A) and was enriched in pathways related to cell cycle regulation, DNA repair, and replication (Fig. 4C). As these pathways are closely associated with cellular proliferation and aging, we assessed senescence using senescence-associated β-gal staining, which confirmed pronounced chondrocyte senescence by W3 and W6, with some positive cells already visible at W1 (fig. S5C).

In contrast, profile 1 (6303 genes) showed a transient decrease during the pre-OA phases (D3 and W1; fig. S5B), involving metabolic processing, transport, and posttranslational protein modification pathways (Fig. 4D). To examine the impact of this temporal down-regulation on ECM structure during incipient OA reprogramming, we conducted gene set enrichment analysis (GSEA) at the pre-OA stage. At D3, it displayed notable suppression of the collagen posttranslational modification pathway (e.g., lysyl-hydroxylated collagen peptides dissociation in the Reactome; fig. S6, A and B). These findings suggest that early disruption of collagen cross-linking potentially represents the molecular basis for compromised ECM integrity at the pre-OA stage.

### Col9 as a pre-OA ECM integrity marker

To elucidate the primary biological activities during the pre-OA stage, we performed Gene Ontology (GO) enrichment analysis of biological processes. This analysis revealed strong involvement of pathways related to “extracellular structure organization” (fig. S6, C and E). This ECM reorganization was mediated through component-binding activities identified by the analysis of molecular functions, such as collagen-binding (fig. S6, D and F). A heatmap of ECM network-related gene expression showed marked down-regulation of multiple transcripts critical for cartilage structure at D3 and W1 (fig. S7). By integrating these data with GSEA (fig. S6B) and differentially expressed genes (DEGs; volcano plots in Fig. 4, E and F), we identified a significant reduction in *Col9*, including *Col9a1* and *Col9a2*, in pre-OA cartilage (*P* < 0.0001). Reverse transcription quantitative polymerase chain reaction (RT-qPCR) confirmed *Col9* down-regulation at D3 and W1 (Fig. 4, G and H). Consistent with the transcriptional findings (Fig. 4, E to H), immunofluorescence analyses confirmed a substantial reduction in Col IX in both fluorescent area and signal intensity at D3 and W1, whereas Col II remained largely unchanged in the MM region (Fig. 4, I to K).

To investigate the role of Col IX in the onset and progression of cartilage degeneration, we performed an in vitro evaluation using a pellet model mimicking OA pathology (fig. S8A). Col IX expression was substantially reduced under both low and high concentrations of interleukin-1β (IL-1β), whereas Col II exhibited only minor changes under low inflammation conditions (fig. S8, B to F), suggesting that Col IX is particularly sensitive to inflammatory stimuli, such as mechano-inflammatory stimuli during OA onset. These findings imply that Col IX depletion during the pre-OA stage may serve as a key marker and therapeutic target for preventing cartilage degeneration by maintaining ECM integrity.

### *Col9* mRNAs attenuate OA progression

**Fig. 5.**
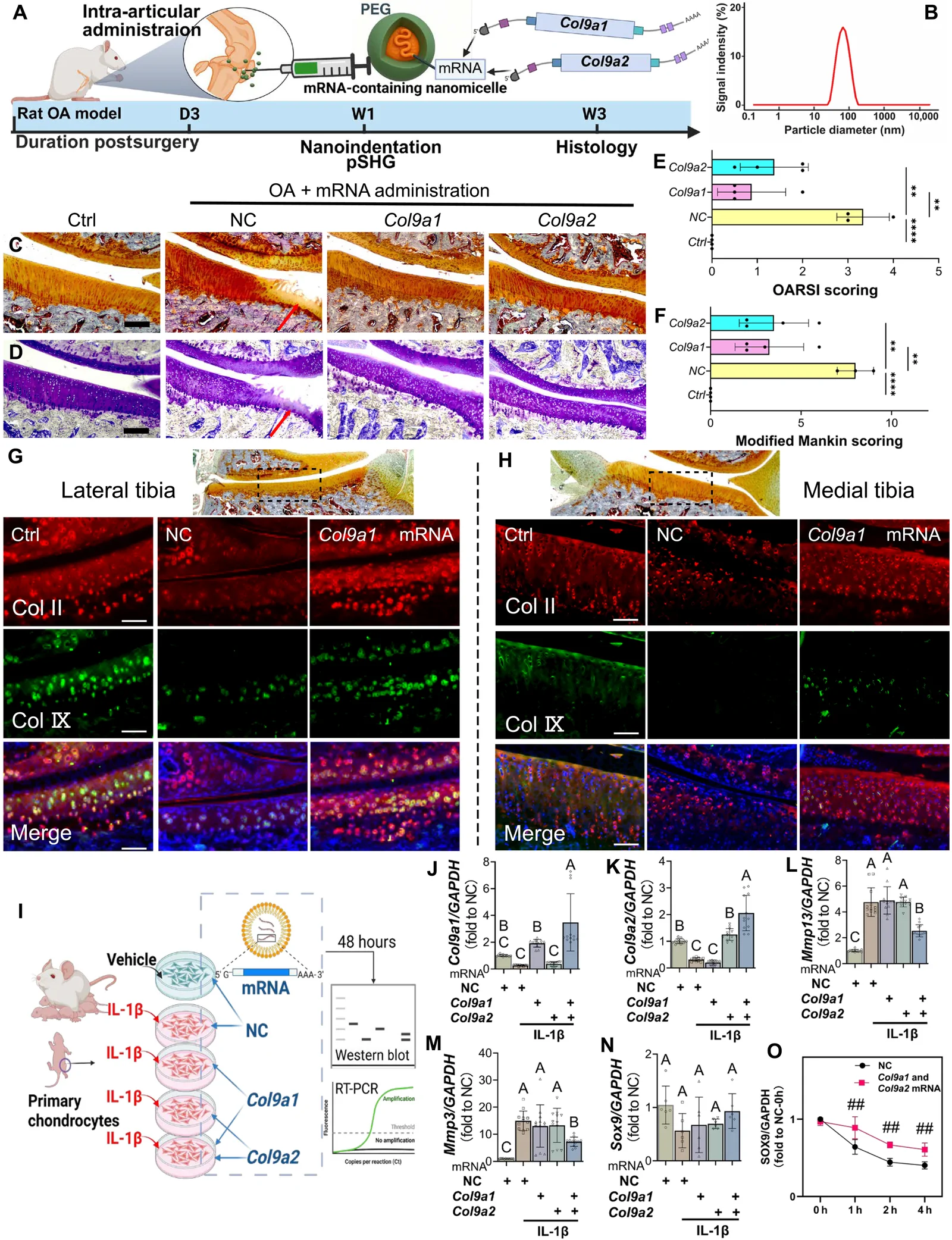
*Col9* mRNAs attenuate OA progression. (**A**) Experimental workflow. *Col9* mRNAs (*Col9a1* and *Col9a2*), synthesized with 5′ CleanCap-3Me capping and complete *N*1-methylpseudouridine substitution, were delivered intra-articularly via polyplex nanomicelles; nontargeted mRNA served as negative control (NC). (**B**) EGFP mRNA–loaded nanomicelles displayed a uniform size distribution (peak diameter below 100 nm). (**C** and **D**) Safranin O/Fast Green (C) and toluidine blue (D) staining of medial tibial cartilage at 3 weeks post-OA following mRNA treatment (scale bars, 200 μm). (**E** and **F**) OARSI (E) and modified Mankin (F) scores evaluating OA progression after mRNA administration (means ± s.d.; *n* ≥ 3). (**G** and **H**) Representative immunostaining of lateral (G) and medial (H) tibial cartilage for Col II (red), Col IX (green), and nuclei (DAPI; blue) at W3 after mRNA administration and OA surgery. Ctrl, age-matched intact cartilage at W3. Merged images show Col II/Col IX colocalization (scale bars, 100 μm). (**I**) Experimental protocol. Chondrocytes were treated with *Col9a1*, *Col9a2*, or both mRNAs, followed by IL-1β stimulation. Cells were collected 48 hours later for gene and protein analyses. (**J** to **N**) RT-qPCR analysis of *Col9a1*, *Col9a2*, *Mmp13*, *Mmp3*, and *Sox9* expression under different treatment conditions (means ± s.d.; *n* ≥ 6; one-way ANOVA followed by Tukey’s multiple comparisons test). Groups not sharing a common letter (A, B, and C) differ significantly (*P* < 0.05). (**O**) Quantification of SOX9 degradation in chondrocytes treated with *Col9a1* and *Col9a2* mRNAs. ##*P* < 0.01 versus NC at the same time point (two-way ANOVA with Tukey’s multiple comparisons test); *n* = 3 (replicate structure in Materials and Methods). (A and I) Created in BioRender. Zhou, J. (2026) https://BioRender.com/nsj2tr5. D3, 3 days; W1, 1 week; W3, 3 weeks; h, hours.

*Col9a1* and *Col9a2* mRNAs were synthesized with 5′ CleanCap-3Me capping and complete *N*1-methylpseudouridine substitution to minimize the innate immunogenicity and administered via intra-articular injection using a polyplex nanomicelle carrier at a dose of 10 μg per 50 μl (Fig. 5, A and B) (*27*). Age-matched intact cartilage without OA induction served as the Ctrl, whereas the nontargeted control (NC) group was treated with *EGFP* mRNA. In the NC group, Safranin O/Fast Green and toluidine blue staining revealed PG depletion and cartilage lesions in the MO region (Fig. 5, C and D, red arrows), resembling changes observed at W3 (Fig. 1, E and F). In contrast, *Col9* mRNA treatment preserved cartilage integrity in the MO region. Both the OARSI (Fig. 5E) and modified Mankin scores (Fig. 5F) demonstrated significant OA progression in NC joints, whereas *Col9* mRNAs suppressed disease progression (*P* < 0.01). Immunofluorescence analyses of Col IX and Col II in both lateral and medial tibial cartilage confirmed the effective delivery of *Col9* mRNAs into dense cartilage (Fig. 5, G and H). In the *Col9a1* mRNA–treated group, higher Col IX expression was observed in the cartilage tissue than in the NC group on both tibia sides. Although Col IX was already depleted at the pre-OA stage, restoration of Col IX by mRNA therapy facilitated the retention of Col II expression (Fig. 5, G and H; and fig. S9, A and B).

To explore the regulatory role of *Col9* in chondrocyte homeostasis, we used an ex vivo model in which primary chondrocytes were treated with *Col9* mRNAs, followed by IL-1β stimulation (Fig. 5I). Cotreatment with *Col9a1* and *Col9a2* mRNAs was used to determine whether the structural integrity of the Col IX complex exerted a synergistic effect on maintaining chondrocyte homeostasis. While IL-1β markedly suppressed endogenous *Col9* in the NC group, *Col9a1* mRNA treatment restored *Col9a1* gene expression (Fig. 5J). Notably, *Col9a2* mRNA alone did not alter *Col9a1* expression, but codelivery of *Col9* mRNAs synergistically increased the expression of both subunits (Fig. 5, J and K). In addition, cotreatment effectively suppressed the catabolic markers *Mmp13* and *Mmp3* (Fig. 5, L and M, and fig. S10A) and restored SOX9 protein levels (fig. S10B), although PCR analyses showed no significant rescue of IL-1β–mediated *Sox9* down-regulation (Fig. 5N). Cycloheximide (CHX) chase assays in IL-1β–stimulated chondrocytes showed that SOX9 protein levels declined more slowly in the cotreatment group than in the NC (fig. S10C and Fig. 5O), indicating that mRNA treatment enhances SOX9 stability and prolongs its half-life compared with that of NC. Collectively, these findings suggest that mRNA therapy targeting *Col9* attenuates OA progression and that the assembly of Col IX subunits maintains chondrocyte homeostasis by stabilizing SOX9 levels.

### *Col9* mRNAs reestablished pre-OA cartilage mechanics and matrix organization

**Fig. 6.**
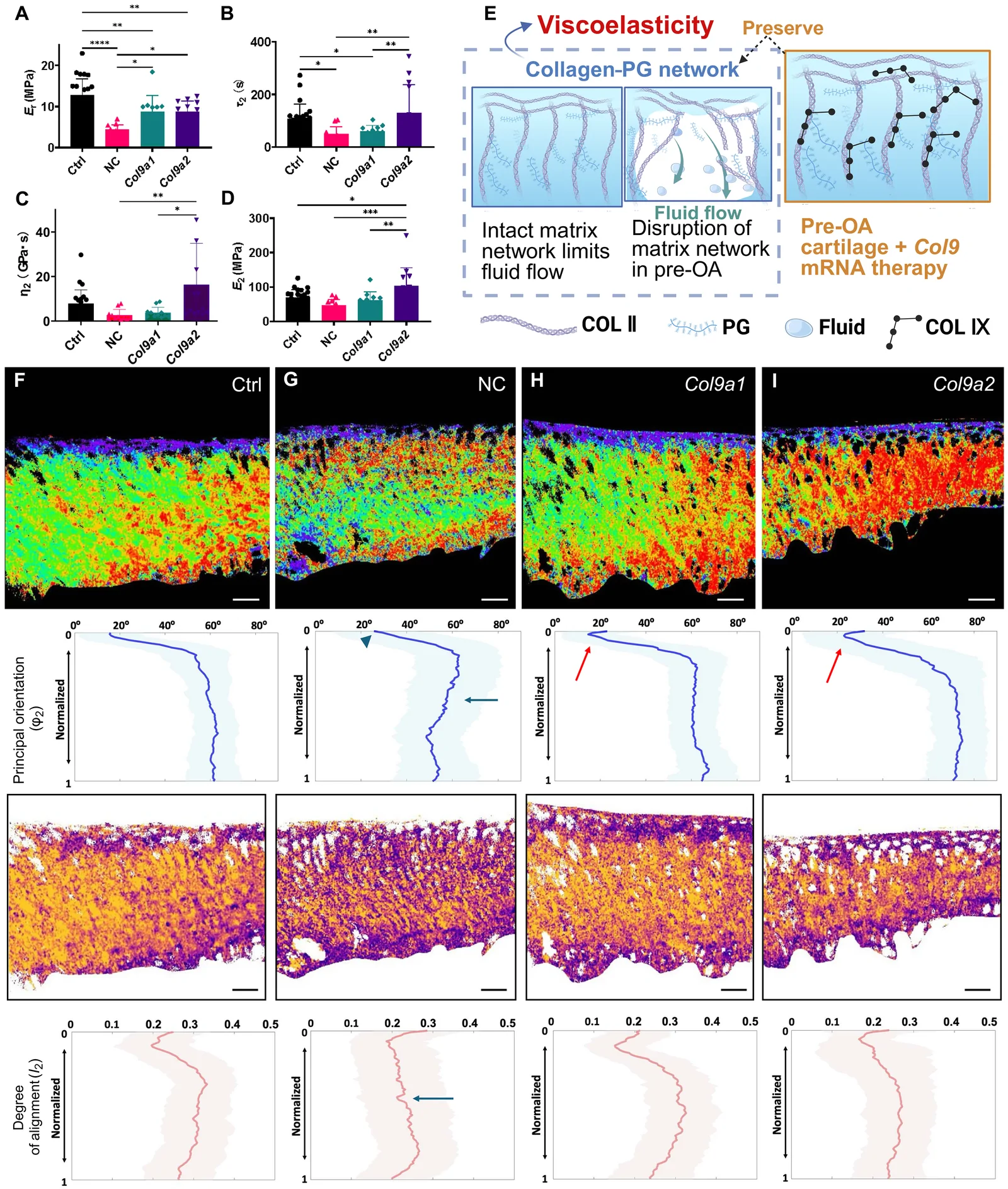
Structural and mechanical characterization of pre-OA cartilage following *Col9* mRNA administration. (**A** to **D**) Mechanical parameters of MM cartilage at 1 week postsurgery, derived from creep loading experiments calculated using the KV model. (A) Reduced elastic modulus (*E*_r_), (B) second retardation time (τ_2_), (C) second viscosity (η_2_), and (D) second elastic modulus (*E*_2_). Ctrl, intact cartilage without OA induction. Data are presented as means ± s.d.; *n* ≥ 3 animals per group; *n* ≥ 3 nanoindentation tests per sample, with > 10 measurements per group. Statistical significance was determined using one-way ANOVA followed by Tukey’s multiple comparisons test (*P < 0.05, **P < 0.01, ***P < 0.001, and ****P < 0.0001). (**E**) Schematic illustrating the role of type IX collagen in preserving cartilage viscoelastic properties by maintaining ECM network integrity. Created in BioRender. Zhou, J. (2026) https://BioRender.com/9p7i9th. (**F** to **I**) Representative pSHG heat maps (512 × 512 pixels, 1 μm per pixel) and corresponding depth-dependent line plots of collagen organization for the Ctrl (F), NC (G), *Col9a1* mRNA (H), and *Col9a2* mRNA (I) treatment groups (*n* = 3). Depth was normalized from the articular cartilage surface to the cement line. NC is the nontarget mRNA as a negative control. The top two rows show the principal orientation of collagen fibrils ($\varphi_{2}$), where colors closer to purple indicate fibrils more parallel to the articular surface, whereas colors closer to red indicate fibrils oriented perpendicular to the articular surface. The bottom two rows present the degree of alignment of collagen fibrils ($I_{2}$), with brighter colors indicating higher fibril alignment. The corresponding line plots summarize the depth-dependent variation in ($\varphi_{2}$) and ($I_{2}$), with solid lines representing mean row values and shaded regions indicating the s.d. within each row.

To determine whether Col IX restoration rescues the integrity in cartilage matrix, we assessed the viscoelastic properties of the MM region using the creep protocol. Compared with intact cartilage, the NC group showed a significantly reduced *E*_r_ (*P* < 0.0001; Fig. 6A), similar to that at W1 (Fig. 2O). The *Col9* mRNA–treated cartilage exhibited marked restoration of *E*_r_ (*P* < 0.05; Fig. 6A). For the first viscoelastic component (τ_1_, *E*_1_, and η_1_), no significant difference in τ_1_ values was observed between NC and Ctrl due to concurrent reduction in both η and *E* (fig. S11). In contrast, the second (main) viscoelastic component (τ_2_) exhibited a significant decline in NC compared with that in Ctrl (*P* < 0.05; Fig. 6B). *Col9a2* mRNA administration preserved the average τ_2_ value at 130.4 s, significantly higher than 50.5 s in NC (*P* < 0.01; Fig. 6B). Both η_2_ (Fig. 6C) and *E*_2_ (Fig. 6D) were higher in the *Col9a2* group than in NC, with a pronounced increase in η_2_, thereby contributing to the preservation of τ_2_. Although the *Col9a1* group showed a moderate increase in τ_2_ (60.8 s), it was not significantly different from that of NC. This mechanical rescue, particularly in terms of viscoelasticity, strongly suggests that Col IX functions as a “molecular staple” (Fig. 6E).

The therapeutic effect of *Col9* mRNA treatment was further supported by analysis of collagen ultrastructural organization using pSHG (Fig. 6, F to I). In NC, substantial structural deterioration was evident, including thinning of the superficial zone (Fig. 6G, blue triangle), loss of vertical alignment and packing order of fibrils in the deep zone (blue arrows), and increased heterogeneity of fibril organization throughout the tissue depth (pink-shaded region shown in Fig. 6G, bottom). *Col9* mRNA treatment preserved superficial zone thickness and structural features (Fig. 6, H and I, red arrows) and maintained the depth-dependent gradient of collagen organization closely resembling that of Ctrl. Collectively, these results demonstrated that *Col9* mRNA therapy mitigates early mechanical dysfunction of the ECM by preventing cartilage structural disorganization during the pre-OA stage.

## DISCUSSION

Articular cartilage bears substantial compressive and shear forces during joint motion. Its unique biomechanical properties arise from a highly organized matrix, with depth-dependent collagen fiber orientation. However, current OA treatments fail to restore these essential mechanical functions. The key challenge lies in replicating the intricate collagen-PG network that underpins long-lasting tissue function. In this study, we found that dysfunction in pre-OA cartilage results from early loss of native-like collagen anisotropy rather than overt matrix degradation. By administering *Col9* mRNA into pre-OA joints, we restored the structure to native-like collagen organization, preserving tissue integrity and preventing degenerative progression.

Using nano-creep interrogation, we clarified the temporal cascade underlying the earliest stages of cartilage dysfunction. This dysfunction originates not only from disruption of the collagen ultrastructure but also from the reduced ability of collagen fibrils to constrain aggrecan, which is essential for fluid-flow resistance, ultimately compromising the unique biomechanical properties of cartilage (Fig. 6E). By W3, the depletion of Col II and PG led to a sharp decline in fluid-flow resistance (Fig. 2G), and the first component of the KV model τ_1_ also decreased. This suggests a critical turning point, distinguishing reversible from irreversible cartilage degradation. Notably, Col IX was already reduced at D3 (Fig. 4K), resulting in altered fibrillar tension of Col II on the cartilage surface and consequently an early decline in *E*_r_, even before major disruption of the collagen network at W1. These biomechanical evaluations reveal a previously unrecognized pre-OA phenotype, in which viscoelastic dysfunction arises from matrix network disruption rather than component loss, aligning with recent discoveries emphasizing the importance of fibril organization in cartilage mechanics (*9*, *28*, *29*).

In this pre-OA state, we further linked structural and mechanical changes to the down-regulation of Col IX, with its early depletion appearing to destabilize the cartilage matrix. Although Col IX was previously considered dispensable in OA pathogenesis (*20*), this conclusion may have stemmed from the relatively mild inflammatory phenotype associated with Col IX deficiency, leaving the potential to modify disease progression largely unexplored. Our mRNA-based treatment restored Col IX expression, guided ECM reorganization, and reproduced the biomechanical function of articular cartilage. These findings suggest that Col9 is both a sensitive early biomarker of OA onset and a proactive therapeutic target capable of reestablishing the anisotropic nature of the ECM before irreversible loss of major matrix components. The role of Col9 is further supported by its function in sustaining the anabolic state of chondrocytes. *Col9* gene expression is regulated by SOX9, a master regulator in chondrocytes (*30*, *31*), and may, in turn, support SOX9 expression, suggesting a positive feedback loop. Structurally, Col IX is composed of a heterotrimer of three distinct α-chains, along with domains with specific functions. These noncollagenous domains protruding into the matrix facilitate Col II fibril cross-linking and maintain proper spacing between fibrils (*32*).

The mRNA modality offers unique advantages for delivering *Col9* during the early stages of OA compared to other alternative Col IX-restoration strategies. To our knowledge, intra-articular delivery of recombinant Col IX protein has not been reported as a viable therapeutic strategy, and, even if administered, free exogenous protein may not be efficiently incorporated into an organized pericellular collagen network. These limitations argue that therapeutic restoration of Col IX must proceed through the induction of endogenous biosynthesis in chondrocytes. Among such strategies, viral vector–based gene therapy represents one candidate approach, but it is associated with immunogenicity and cytotoxicity (*33*), and genomic integration poses an additional concern by precluding precise control over expression duration. In contrast, *Col9* mRNA provides transient, nonintegrating expression that drives Col IX biosynthesis for interfibrillar network stabilization. When Col II fibrils are partially disrupted, *Col9* mRNA can provide therapeutic proteins to maintain matrix structure and mechanical properties. Col IX overexpression restored network tension via mRNA therapy (Fig. 6A) and recovered superficial-zone collagen orientation (Fig. 6, H and I, red arrows), highlighting a critical molecular staple function. Moreover, the successful rescue of mechanical dysfunction at the pre-OA stage by *Col9* mRNA therapy demonstrates that molecular-level intervention can preserve tissue-level function, with the synergistic effect of coadministering *Col9a1* and *Col9a2* mRNAs, emphasizing the importance of maintaining the complete Col IX heterotrimeric structure.

Several aspects merit further investigation to advance the clinical translation of our findings. First, detecting pre-OA mechanical changes in human joints remains a major challenge. Fiber optic or arthroscopic mechanical mapping techniques, such as SHG (*16*), Brillouin scattering (*34*), and optical coherence elastography (*35*), are promising but remain invasive and require further refinement. Second, the animal OA model used in this study involves meniscal removal, inducing sustained mechanical overload. If the underlying pathophysiologic mechanisms closely resemble the chronic, progressive nature of human OA, then administration of *Col9* mRNA at the pre-OA stage may effectively prevent OA onset and repair cartilage much closer to intact tissue. Translation of this concept will require future clinical trials for validation. Last, for cases where OA has already progressed, a combined therapeutic strategy should be explored. *Col9* mRNA therapy could preserve existing Col II architecture, while coadministration with complementary mRNAs may synergistically attenuate disease progression.

In conclusion, this study demonstrates that early viscoelastic dysfunction of cartilage, driven by collagen network disruption, serves as both an indicator and a therapeutic target in pre-OA. We show that fortifying the cartilage matrix through *Col9* mRNA–mediated “matrix stapling” preserves mechanical function and inhibits cartilage degeneration. These findings support a proactive approach to joint degeneration, which not only aims to repair cartilage after failure but also seeks to preempt failure by reinforcing the native structure and function of the tissue. Our study highlights *Col9* mRNA therapy as a promising translational strategy for preventing OA at its earliest stage. Our integrated approach, combining mechanical testing, biophotonic imaging, and molecular therapy, provides a comprehensive framework for developing early interventions.

## MATERIALS AND METHODS

### Experimental design

This study was designed to define previously unrecognized biomechanical and ultrastructural hallmarks of pre-OA cartilage and to investigate whether restoring Col IX via mRNA therapy can preserve cartilage matrix integrity and prevent OA progression. We used an instability-induced rat OA model generated by MMx in 8-week-old female Sprague-Dawley (SD) rats. Disease progression was characterized longitudinally using complementary readouts in the MM region of tibial cartilage. Cartilage degeneration was assessed by histology. The biomechanics in the cartilage matrix were quantified by nanoindentation-based mechanical testing to capture elastic and time-dependent viscoelastic parameters, and collagen network organization was evaluated by pSHG microscopy. Early molecular changes were profiled by RNA-seq and pathway enrichment analyses, followed by RT-qPCR and immunofluorescence validation of key ECM regulators, including Col9 depletion during the pre-OA stage (D3 and W1). To evaluate therapeutic potential, *Col9a1* and *Col9a2* mRNAs were delivered intra-articularly using a polymer-based polyplex nanomicelle. Complementary ex vivo experiments used primary chondrocytes treated with *Col9* mRNAs followed by IL-1β stimulation to evaluate cartilage homeostasis and catabolic responses. Sample sizes, biological replicates, and statistical methods are provided in the corresponding figure legends; histological and image-based analyses were performed blinded where applicable.

### Animal studies

All animal experiments were conducted according to the protocols approved by the Institutional Animal Care and Use Committee of Institute of Science Tokyo (protocol no. A2022-028C5). Animals were housed in standard cages under 12-hour light/dark cycles, with ad libitum access to food and water. An OA model was established in 8-week-old female SD rats (Sankyo Labo Service Corp., Tokyo, Japan) using MMx performed by transecting the medial collateral ligament (MCL), followed by removal of the medial meniscus, as previously described (*22*, *36*). Briefly, under general anesthesia, the MCL of the right knee joint was transected to expose the medial meniscus, which was then fully excised at its narrowest region. The rats were randomly allocated into six groups based on postsurgery duration. The right knee joints of 8-week-old SD rats served as intact controls (Ctrl), whereas the 18-week postoperative group exhibited severe joint damage characteristic of late-stage OA. The rats were euthanized at designated endpoints, and the right knee joints were collected for further analysis.

### μCT and histological analysis

Knee joints were fixed in 4% paraformaldehyde and subjected to μCT using an inspeXio SMX-100CT system (SHIMADZU, Kyoto, Japan). Scans were performed at a voxel resolution of 20 μm for optimal visualization of calcified tissues. Three-dimensional reconstructions and quantitative analyses were performed using 3D-Bon software (RATOC, Japan).

For histological analysis, joint samples were processed for cryosectioning following the method described by Kawamoto and co-workers (*37*, *38*). Sections (4 μm thick) were obtained using a cryostat (CM3050S, Leica Biosystems). Safranin O/Fast Green staining and 0.05% toluidine blue staining were performed to assess cartilage morphology, structure, and ECM composition. Cartilage lesions were semiquantitatively evaluated using the OARSI scoring system (0 to 6) and the modified Mankin scoring system (0 to 16) (*24*). The scores were independently evaluated by two blinded researchers, and mean values were calculated for each slide. Higher scores indicated more severe OA pathology. For regional analysis, the cartilage of the medial tibial plateau was divided into three equal-width zones—the medial inner, MM, and MO zones (from the inner to the outer region)—based on ruler measurements from photographic images (*24*). These regions correspond to the black dashed lines in Fig. 1G.

### Immunofluorescence and β-galactosidase staining

Frozen sections were washed with phosphate-buffered saline (PBS), blocked with 5% normal goat serum in PBST (PBS and 0.1% Tween 20) for 1 hour, and incubated overnight at 4°C with the following primary antibodies: Col II (1:100, Santa Cruz Biotechnology, sc-34712) and Col IX (1:500, gift from F. Zaucke, Goethe University). Fluorophore-conjugated secondary antibodies were applied the following day, and 4′,6-diamidino-2-phenylindole (DAPI) was used to visualize the cell nuclei. Images were captured using a fluorescence microscope (BZ9000, Keyence) and analyzed in Fiji-ImageJ software. Senescent chondrocytes were identified using a Senescence β-Galactosidase Staining Kit (Cell Signaling Technology, no. 9860) following the manufacturer’s protocol (*39*).

### Nanoindentation analysis of cartilage instantaneous modulus

Freshly harvested right tibias were embedded in dental polymethyl methacrylate (Aislar Kulzer), with the joint surface oriented horizontally. The soft tissues (meniscus, ligaments, and synovium) were carefully removed to expose the articular cartilage surface. Nanoindentation tests were performed using a TI 950 TriboIndenter (Hysitron Inc., Eden Prairie, MN, USA) equipped with an atomic force microscope. A diamond flat-end indenter (10-μm diameter) was used to measure the instantaneous reduced modulus of the cartilage. Indentations were made in the MM and MO regions just adjacent to the medial tibial plateau. Two independent load functions were applied at separate indentation points within the MM zone: (i) a rapid unload protocol for *E*_inst_ determination and (ii) a creep-loading protocol for generalized KV model fitting. Throughout testing, the specimens were kept hydrated with PBS. Force-displacement curves were recorded under loading/unloading rates of 50 μN/0.1 s, with a maximum force of 50 μN. A 1-s hold at maximum force was applied between loading and unloading, followed by a rapid unloading phase of 50 μN/0.1 s. An additional 60-s hold was included during the unloading segment at 2% of the maximum force. The purpose of this loading protocol was to discern the instantaneous elastic response and delayed viscoelastic behavior as the viscous dashpot cannot respond immediately under high strain rates. The instantaneous force drop during unloading (Δ*F*′) corresponds to a nearly pure elastic response and subsequent time-dependent force reduction (Δ*F*″). The detected Δ*F*′ corresponds to elastic deformation (Δ*h*′) leading to the calculation of the instantaneous elastic modulus (*E*_inst_) according to the stress (δ)–strain (ε) relationship (*E* = δ/ε). In this study, strain was determined as ε = Δ*h*′/*D*, where *D* is the indenter-tip diameter (10 μm) associated with the classical Boussinesq solution for viscoelastic solids (*40*). To ensure consistency, all measurements were conducted at room temperature (22° ± 1°C), and the indenter tip was calibrated before testing.

### Creep analysis using generalized KV models

A creep loading protocol was used to isolate the intrinsic ECM viscoelasticity of the cartilage. The loading protocol consisted of a rapid loading phase (0.1 s) up to a maximum load of 5 μN with a loading rate k, followed by a 35-s hold phase and a subsequent rapid unloading phase (0.1 s). This approach minimizes the effects of fluid flow and enables quantification of the solid matrix viscoelastic response. The viscoelastic behavior of the cartilage was analyzed using the generalized KV model (*25*, *26*). Briefly, for a viscoelastic material under indentation with a flat-end indenter, the total deformation can be expressed as follows (*25*)

$$h_{\text{total}}=h_{\text{visc}}+h_{e}+h_{p}=\frac{1-\upsilon }{2R}\cdot \frac{P}{2G}$$ (1)

where *P* denotes the applied load; $h_{\text{total}}$, $h_{\text{visc}}$, $h_{e}$, and $h_{p}$ denote the total deformation and the deformations contributed by the viscous and elastic behavior of the cartilage tissue, respectively; ν is the Poisson’s ratio; *G* is the shear modulus; and *R* is the indenter tip radius. Specifically, $\frac{P}{2G}$ is a deformation-related quantity determined using a hereditary viscoelastic integral operator for creep

$$\frac{P}{2G}=\int_{0}^{t}J\left(t-t^{\prime }\right)\frac{\mathrm{dP}}{{\mathrm{dt}}^{\prime }}{\mathrm{dt}}^{\prime }$$ (2)

where $t^{\prime }$ is the dummy variable for time integration and J(t) is the creep function of the materials. Based on our preliminary trial-and-error approach (*14*), the generalized KV model, comprising one pure elastic time-independent element and two KV bodies (fig. S12), was identified as a suitable rheological model for describing the creep behavior J(t), which can be expressed as

$$J\left(t\right)=\frac{1}{E_{0}}+\sum_{i=1}^{2}\frac{1}{E_{i}}\left[1-\text{exp}\left(\frac{-t}{\tau_{i}}\right)\right]$$ (3)

where $E_{=i}$(i=0,1,2) are the elastic component, and $\tau_{i}=\frac{\eta_{i}}{E_{i}}$ (i=1,2) represent the viscosity-related components of the elements. Following simplification and organization of Eqs. 1 to 3, the *h*-*P* relationship can be expressed as (*25*)

$$h_{\text{total}}=\left\{ \begin{array}{ll} m_{0}^{*}\cdot t+\sum_{i=1}^{2}m_{i,1}^{*}\left[\text{exp}\left(-m_{i,2}^{*}\cdot t\right)-1\right]; & \text{Loading period}\\ m_{0}^{*}-\sum_{i=1}^{2}m_{i,1}^{*}\text{exp}\left(-m_{i,2}^{*}\cdot t\right); & \text{Holding period} \end{array}\right.$$ (4)

where $m_{0}^{*}$ is the generalized parameter for the elastic element and $m_{i,\;1}^{*}$ and $m_{i,\;2}^{*}$ (*i* = 1,2) are the generalized parameters for the two viscous elements

$$\left\{ \begin{array}{l} m_{0}^{*}=\frac{1-\upsilon }{2R}\cdot k\cdot \left(\frac{1}{E_{0}}+\frac{1}{E_{1}}+\frac{1}{E_{2}}\right)\\ m_{i,\;1}^{*}=\frac{1-\upsilon }{2R}\cdot k\cdot \frac{\tau_{i}}{E_{i}};\;\left(i=1,2\right)\\ m_{i,\;2}^{*}=\frac{1}{\tau_{i}};\;\left(i=1,2\right) \end{array}\right.$$ (5)

Furthermore, $m_{0}^{*}$, $m_{i,1}^{*}$, and $m_{i,\;2}^{*}$ were estimated by least-squares fitting of the raw data during the holding period to Eq. 4, thereby determining the rheological parameters of the samples. Subsequently, the purely elastic responses can be mathematically distinguished by subtracting the calculated viscoelastic contribution from the overall mechanical behavior, according to Eq. 1. For brevity, complete derivation is omitted here but can be found in our previous work (*14*).

The obtained retardation parameters ($\tau_{i}$) quantify the time-dependent viscous response. The time-independent elasticity (i.e., the reduced modulus, *E*_r_) can be analyzed using the Oliver-Pharr method (*41*) based on the unloading curves of the distinguished pure elastic response. This analysis allows for a clear separation between the viscoelastic and elastic components of the cartilage mechanical response, providing insight into its time-dependent behavior under load.

### Chondrocyte isolation and micromass culture

Chondrocytes were obtained from the knee joints of 3-day-old SD rats through sequential enzymatic digestion, as described previously (*42*). Briefly, knee cartilage tissue was excised after complete removal of the synovium and other tissues and then immersed in PBS. The cartilage tissue was minced into small fragments and sequentially digested with 0.25% trypsin for 30 min, followed by 0.25% type II collagenase (Worthington) at 37°C for 6 hours. After filtration through a 70-μm filter, chondrocytes were collected by centrifugation at 1500 rpm for 5 min and cultured in Dulbecco’s modified Eagle medium/F-12 (DMEM/F-12; Wako, Japan) supplemented with 10% fetal bovine serum (Funakoshi, Japan) and 1% penicillin/streptomycin (Sigma-Aldrich, USA) at 37°C in an incubator containing 5% CO_2_. After cell passaging, chondrocytes within passage 3 were used for experiments. To obtain OA-like phenotypes, chondrocytes were treated with recombinant rat IL-1β protein (1 and 10 ng/ml; R&D Systems, USA).

Micromass culture was performed as described previously (*43*). Briefly, passage 1 chondrocytes were seeded at a density of 2.5 × 10^5^ cells in 10-μl droplets onto 24-well plates. Chondrocytes were treated with or without IL-1β (10 ng/ml) for 7 days, and the culture medium was replaced every 2 days. The ECM PG content was evaluated using Alcian Blue staining (Sigma-Aldrich).

### Gene expression analysis

Total RNA was extracted from the tibial cartilage of injured joints using TRIzol reagent (Invitrogen, USA) according to the manufacturer’s instructions. Total RNA (1 μg) was used to synthesize cDNA with the ReverTra Ace qPCR RT kit (Toyobo, Japan). The mRNA levels of each gene were normalized to *Gapdh* within the same sample, and the relative expression of target genes was calculated using the formula described by Livak and Schmittgen (*44*). RT-qPCR was conducted using PowerTrack SYBR Green Master Mix (Applied Biosystems, Foster City, CA, USA) on a StepOnePlus Real-time PCR system (Applied Biosystems). The primers used for quantitative analysis are listed in table S1.

### Bulk RNA-seq and data processing

Transcriptome sequencing of total RNA samples from *Rattus* was performed by excising cartilage from injured joints. Stranded libraries with rRNA depletion were prepared using standard protocols and sequenced on the Illumina NovaSeq platform (Azenta Japan Corp.). Adapters and low-quality reads (*Q* value ≤ 20) were trimmed and removed using fastp (v0.23.1) (*45*). High-quality, clean paired-end reads were mapped to the *Rattus norvegicus* reference genome (Ensembl release 104) using HISAT2 v2.2.1 (*46*) with default parameters. After sorting with SAMtools (v1.13) (*47*), reads were assigned to genes using featureCounts (v2.0.1) (*48*) to obtain the raw counts for each gene. Normalized gene expression levels were calculated as fragments per kilobase of transcript per million mapped reads.

PCA was performed on the total gene expression dataset to assess the overall differences among groups (*49*). Differential expression analysis was performed using DESeq2 (v1.32.0) (*50*) based on the raw count data. DEGs were identified using the differences in expression levels (|log_2_ fold change| > 1 and adjusted *P* value < 0.05, Benjamini-Hochberg correction) among the samples (*51*). To identify the longitudinal gene expression patterns during OA progression, the STEM method was applied, which clusters genes according to their significance and expression patterns (*52*). For functional enrichment analysis, WebGestalt (WEB-based GEne SeT AnaLysis Toolkit, www.webgestalt.org/) was used (*53*). The list of DEGs was uploaded to WebGestalt, and enrichment analysis was performed for the Reactome pathways and GO categories. GSEA was performed using GSEA software (v.4.3.3) to determine the hallmark gene set.

### Western blotting and CHX chase assay

Protein extraction and immunoblotting were performed as previously described (*42*). Briefly, total protein was isolated using radioimmunoprecipitation assay buffer following the manufacturer’s instructions. Equal amounts of protein (20 μg per lane) were separated by SDS–polyacrylamide gel electrophoresis and transferred onto polyvinylidene difluoride membranes. After blocking with 5% bovine serum albumin, the membranes were incubated with primary antibodies overnight at 4°C, followed by three 15-min washes with tris-buffered saline containing 0.1% Tween 20 (TBST). The membranes were then incubated with horseradish peroxidase–conjugated secondary antibodies for 1 hour at room temperature and washed again with TBST. Protein bands were visualized using an enhanced chemiluminescence substrate (no. 32106, Thermo Fisher Scientific, USA) and imaged with a Bio-Rad imaging system (Bio-Rad). Band intensities were quantified using ImageJ software.

For the CHX chase assay, primary chondrocytes were transfected with *Col9a1* or *Col9a2* (Elixirgen Scientific Japan Inc., Japan) or codelivered *Col9* mRNAs using Lipofectamine MessengerMAX transfection reagent. Transfected cells were then cultured for 48 hours in the presence of IL-1β (10 ng/ml). After medium replacement and a 1-hour incubation, CHX was added at a final concentration of 100 μg/ml to inhibit protein synthesis. Cells were collected at 0, 1, 2, and 4 hours after CHX treatment for protein stability analysis. SOX9 protein levels were measured using western blotting to assess degradation kinetics.

For the CHX Western blots (Fig. 5O and fig. S10C), lysates from three independent biological replicates per condition were pooled in equal volumes, and three aliquots of this pool were each electrophoresed and immunoblotted on separate occasions. For the SOX9, MMP13, and MMP3 Western blots (fig. S10A), lysates from biological replicates per condition were pooled, and the membranes were sequentially probed for SOX9, MMP13, MMP3, and GAPDH. This pooled-lysate approach was used to assess trend reproducibility for primary chondrocyte cultures. Densitometry from the resulting three sample sets per condition is reported as *n* = 3 in Fig. 5O and fig. S10B.

### pSHG microscopy analysis

The embedded samples (*n* = 3 per group) were imaged using a custom-modified multiphoton confocal microscope (Olympus Fluoview 300 BX51, Olympus Corp., Tokyo, Japan) at the MM regions. The central excitation wavelength was set to 810 nm using a mode-locked femtosecond Ti:Sapphire laser (Mira 900-D, Coherent Inc.). A water-immersion, long working-distance objective lens (Olympus XLPLN25XWMP, numerical aperture of 1.05) was used to collect the backscattered light emitted by the sample. The scanning configuration was adjusted to acquire images with a field of view of 512 × 512 pixels about 1 μm in size. pSHG imaging was performed using a half-wave plate (AHWP05M-980, Thorlabs, USA) and a quarter-wave plate (AQWP05M-980, Thorlabs, USA) to vary the polarization angle of the incident light from 0° to 176°. Images were acquired at 12 equally spaced polarization angles, and the 12 signal intensities at the same pixel were fitted with a circular function (Eq. 6) using the least-squares method (*15*, *54*, *55*), where α represents the linear polarization angle relative to the horizontal axis of the sample (parallel to the articular surface in our setup). The Fourier coefficients obtained from the fitting function (Eqs. 7 and 8) were used to derive the degree of alignment ($I_{2}$) of collagen fibrils and their principal orientation ($\varphi_{2}$), respectively

$$I\left(\alpha \right)=a_{0}+a_{2}\text{cos}\left(2\alpha \right)+b_{2}\text{sin}\left(2\alpha \right)+a_{4}\text{cos}\left(4\alpha \right)+b_{4}\text{sin}\left(4\alpha \right)$$ (6)

$$I_{2}=\frac{\sqrt{a_{2}^{2}+b_{2}^{2}}}{a_{0}}$$ (7)

$$\varphi_{2}=\frac{1}{2}{\text{tan}}^{-1}\left(\frac{b_{2}}{a_{2}}\right)$$ (8)

$I_{2}$, as a unitless parameter, can be used as an indicator of the degree of alignment of collagen fibril (as opposed to the degree of dispersion). A higher $I_{2}$ value indicates more uniformly aligned fibrils and stronger directionality within a given pixel. Meanwhile, $\varphi_{2}$ denotes the principal orientation of the collagen fibrils relative to the horizontal axis (x).

A binary mask was first applied to exclude the subchondral bone based on the distinct collagen textures across the cement line. The heat maps of $I_{2}$ and $\varphi_{2}$ were then used to illustrate the local spatial patterns of the collagen organization. To better visualize the depth-dependent trend, each map was converted into a line plot by averaging the pixel values along each row and normalizing the depth from the articular cartilage surface to the cement line. The solid lines represent mean row values, and the shaded region indicates the standard deviation (s.d.) of the pixel values within each row, reflecting the lateral heterogeneity of the collagen organization across the tissue depth.

### Intra-articular injection of mRNAs encoding *Col9a1* or *Col9a2*

Intra-articular mRNA injection was performed as previously described (*22*). mRNAs encoding *Col9a1* (NM_001100842.1) or *Col9a2* (NM_001108675.1) were synthesized by Elixirgen Scientific Japan Inc. (Japan), with the 5′ end capped using CleanCap-3Me and complete substitution by *N*1-methylpseudouridine (m1Ψ). The mRNA quality was verified using an Agilent 2100 Bioanalyzer (Agilent Technologies, Santa Clara, CA, USA) (fig. S13). mRNAs were injected into the joint using a polymer-based carrier, a polyplex nanomicelle. Briefly, a block copolymer, polyethylene glycol–poly(*N*-{*N*′-[*N*″-(2-aminoethyl)-2-aminoethyl]-2-aminoethyl} aspartamide) [PEG-PAsp(TET)], was synthesized as previously described (*27*). The molecular weight of PEG was 12 kDa, and the polymerization degree of PAsp(TET) was 63, as determined by ^1^H nuclear magnetic resonance analysis (JEOL EX300 spectrometer, JEOL, Tokyo, Japan). To prepare mRNA-loaded polyplex nanomicelles, mRNA and the block copolymer were separately dissolved in 10 mM Hepes buffer (pH 7.3) and then mixed by adjusting the N/P ratio (the residual molar ratio of the polycations in the amino groups to the mRNA phosphate groups) to 8. The resulting nanomicelles had a diameter of ∼90 nm with an almost neutral surface charge, as measured by a Zetasizer Nano ZS (Malvern Instruments Ltd., Worcestershire, UK) (Fig. 5B). A total of 50 μl of the nanomicelle solution containing 10 μg of mRNA was injected through the patellar tendon using a 30-gauge syringe under anesthesia.

### Statistical analysis

Statistical analyses were performed using GraphPad Prism v.10.2.3 (GraphPad Software, San Diego, CA, USA). Differences among multiple groups were assessed using two-way analysis of variance (ANOVA) followed by Tukey’s multiple comparisons test. For single-factor comparisons involving more than two groups, one-way ANOVA with Tukey’s post hoc test was applied. Data are presented as the means ± s.d. Box plots show the data distribution, where boxes indicate the interquartile range (IQR), the central line represents the median, and whiskers extend to 1.5 times the IQR from the first and third quartiles. Outliers were identified and plotted individually. Groups that do not share a common letter in the figures (e.g., A, B, and C) were considered significantly different (*P* < 0.05), as determined by Tukey’s multiple comparisons test.
