# Supplementary material for: Restoring pre-osteoarthritis cartilage matrix integrity via Col9 mRNA halts osteoarthritis progression
Source: Sci Adv. 2026 Sep 2;12(36):eaef9739. doi: 10.1126/sciadv.aef9739 (PMC13537250; doi:10.1126/sciadv.aef9739)
Supplement: Supplementary file 1 [file sciadv.aef9739_sm.pdf]

Science Advances
AAAS

# Supplementary Materials for

## Restoring pre-osteoarthritis cartilage matrix integrity via Col9 mRNA halts osteoarthritis progression

Jun Zhou *et al.*

Corresponding author: Keiji Itaka, itaka@cider.osaka-u.ac.jp

*Sci. Adv.* **12**, eaef9739 (2026)
DOI: 10.1126/sciadv.aef9739

**This PDF file includes:**

Figs. S1 to S13
Table S1

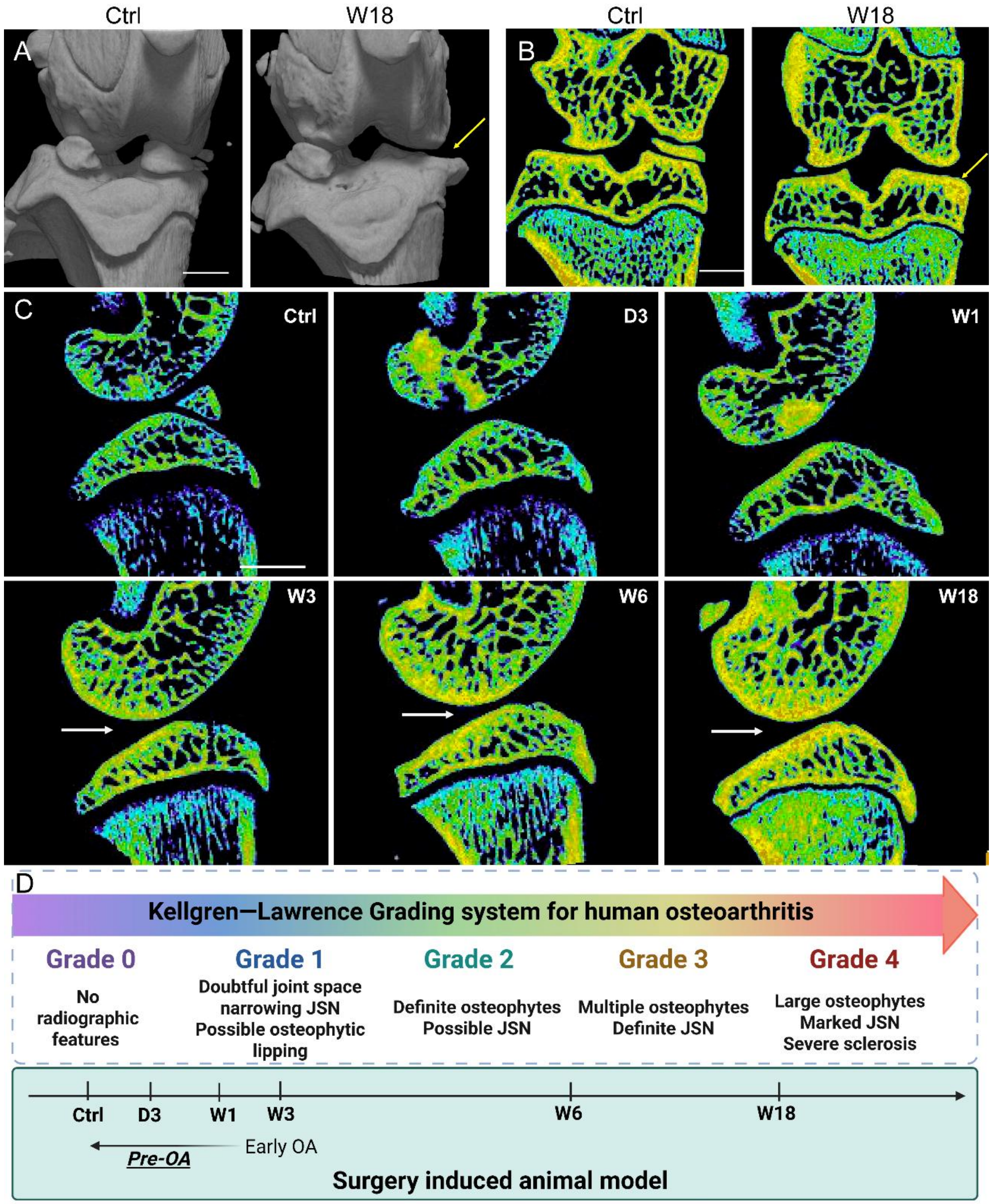


**Fig. S1. Representative μCT analysis of subchondral bone and clinical relevance.**
(**A**) Representative μCT 3D images at 18 weeks post-surgery (W18) and age-matched intact joints (26 weeks old, Ctrl). (**B**) Representative coronal sections of the femurs and tibias. Yellow arrows indicate osteophytes. (**C**) Representative sagittal μCT sections of the femurs and tibias. White arrows indicate joint space narrowing (JSN). Scale bar: 2 mm. (**D**) Comparison of rat osteoarthritis (OA) progression with human clinical OA classification based on the Kellgren–Lawrence grading system. Panel **D** was created in BioRender. Zhou, J. (2026); https://BioRender.com/buacq26.

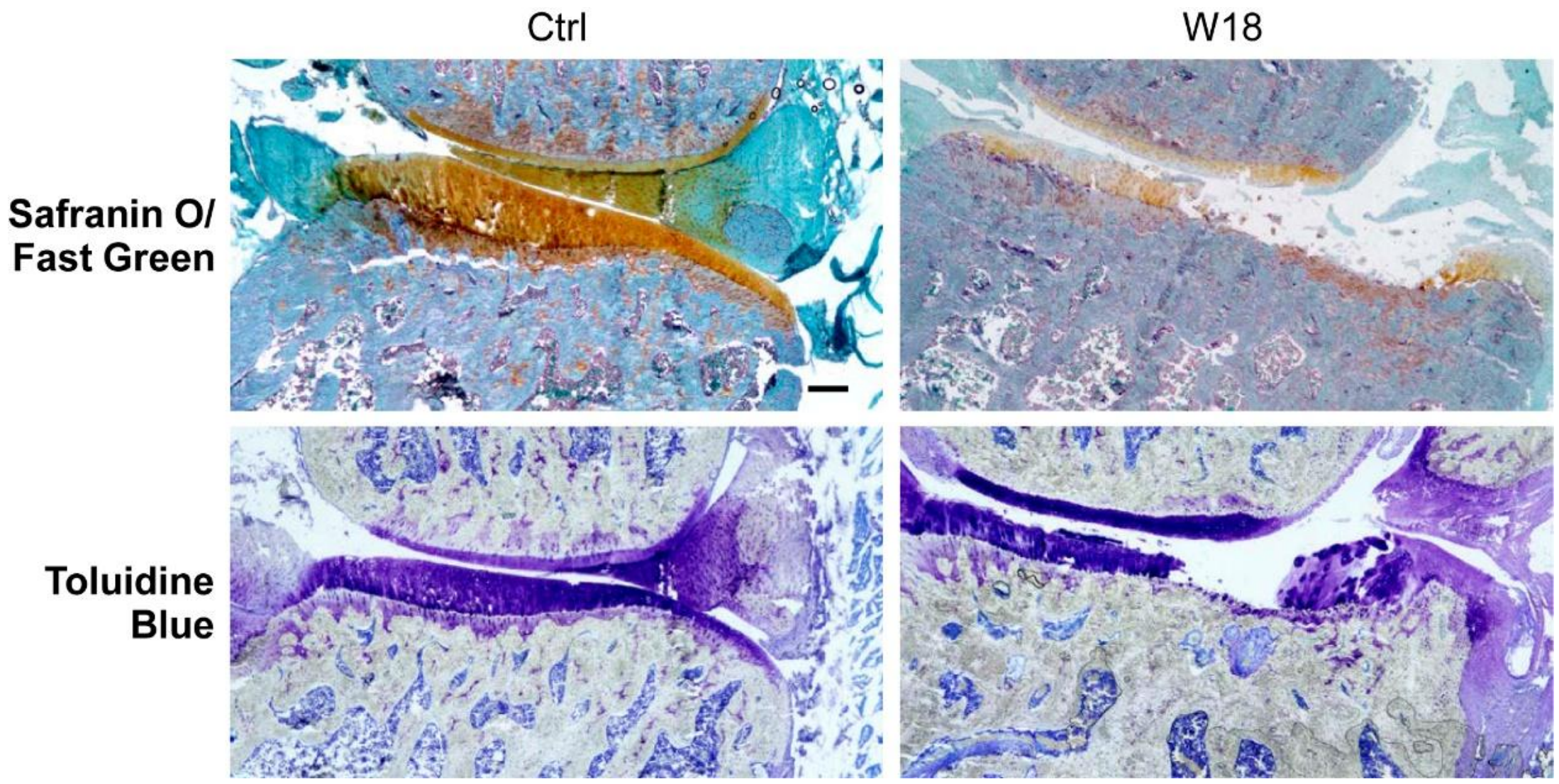


**Fig. S2. Representative histology of late-stage osteoarthritis (OA) cartilage.**

Representative Safranin O/Fast Green and Toluidine Blue staining of cartilage 18 weeks post-OA surgery (W18) compared with age-matched controls (26 weeks old, Ctrl). Scale bar: 200 µm.

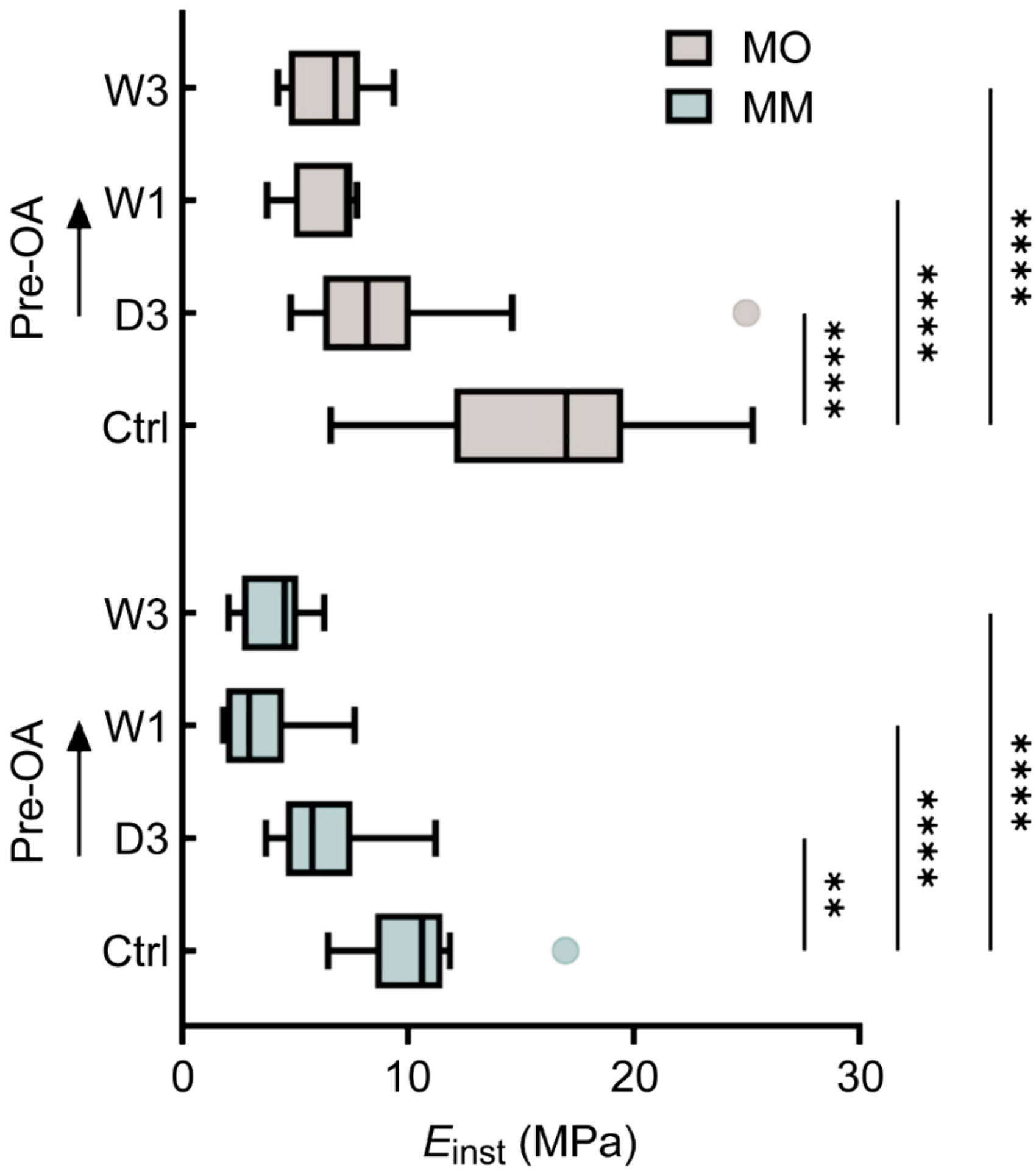


**Fig. S3. Alterations in the instantaneous modulus of cartilage during early osteoarthritis (OA).**

Box plot showing the instantaneous modulus ($E_{inst}$) measured across different OA time points in both the medial middle (MM) and medial outer (MO) regions. Boxes represent the interquartile range (IQR), with the line inside each box indicating the median. Whiskers extend to the smallest and largest values within 1.5 times the IQR from the first and third quartiles, respectively. Data points outside this range are considered outliers and are plotted individually. n ≥ 3/group, with 1–5 measurements per sample. Statistical significance was determined using one-way ANOVA followed by Tukey's multiple comparisons test (**$p < 0.01$, ***$p < 0.001$, ****$p < 0.0001$).

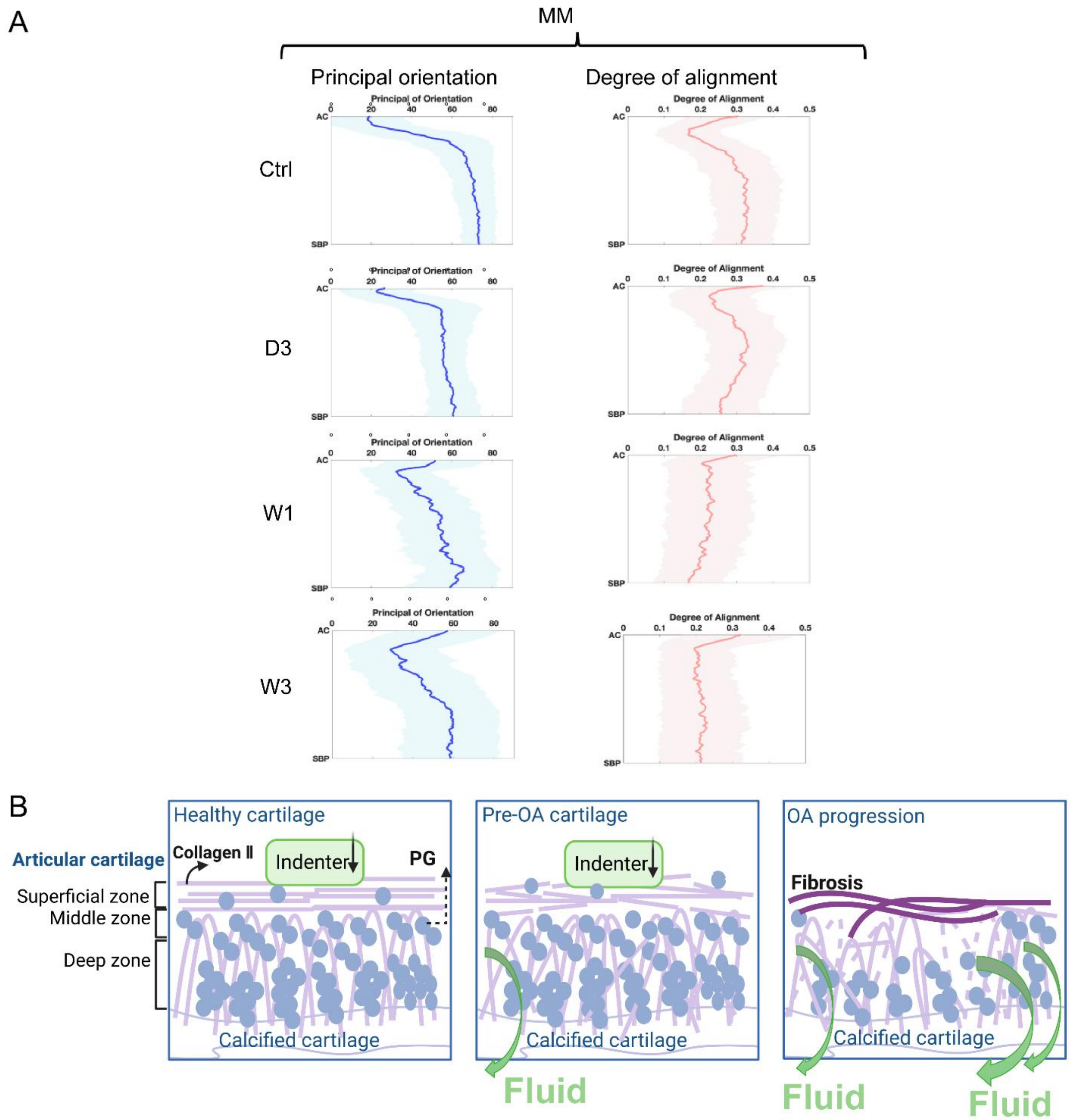


**Fig. S4. Ultrastructural disorganization of collagen fibrils in the medial middle (MM) region during early osteoarthritis (OA).**
(**A**) Representative curves of organization change in collagen fibrils evaluated by polarization-resolved second harmonic generation microscopy from 3 days post-OA (D3) to 3-week post-OA (W3) compared with intact joint (Ctrl). (**B**) Schematic illustration of mechanical changes and collagen network reorganization during the transition from pre-OA to OA. Created in BioRender. Zhou, J. (2026); https://BioRender.com/lm99x60.

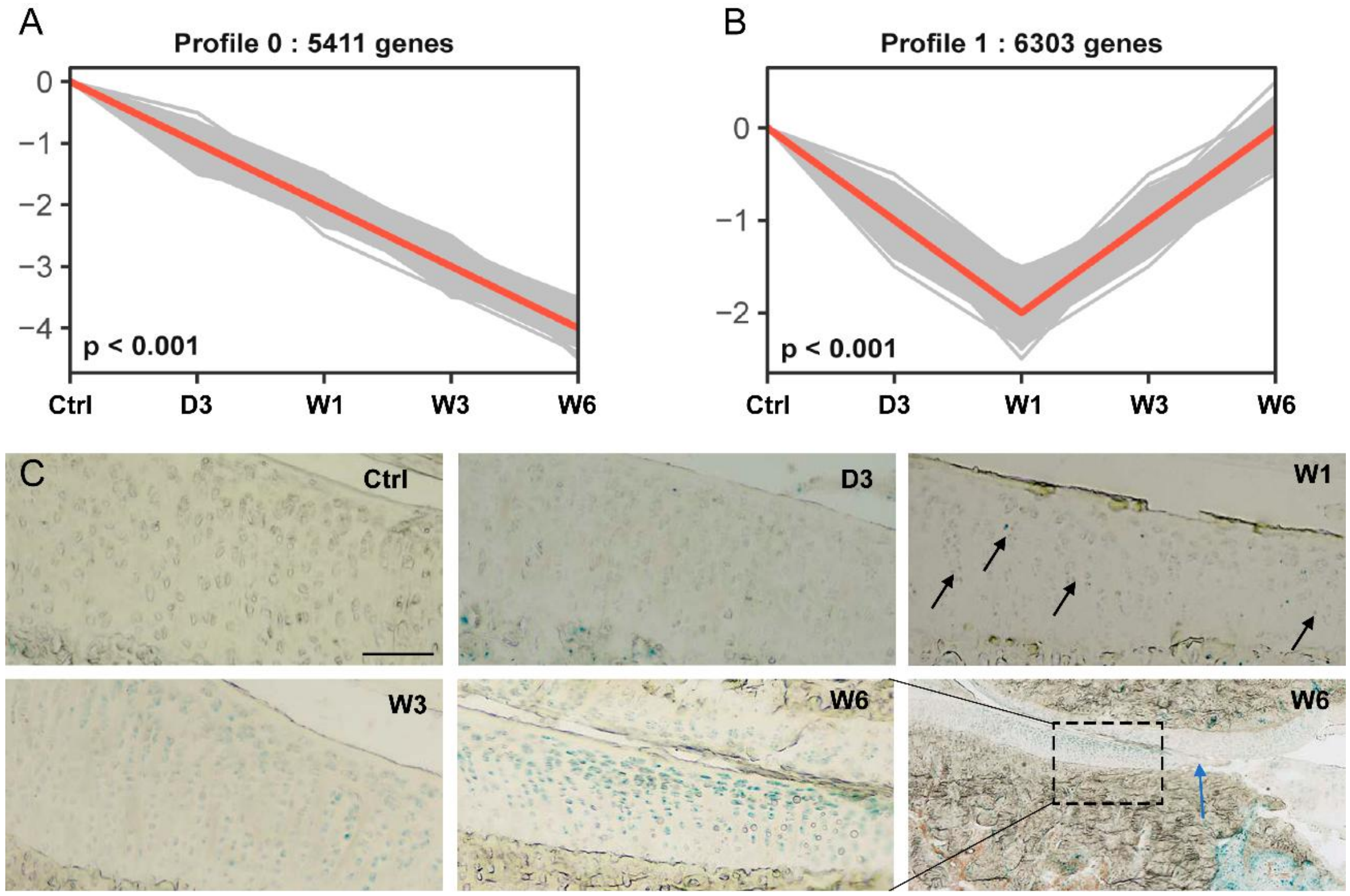


**Fig. S5. Transcriptomic profiling and chondrocyte senescence during osteoarthritis (OA) progression.**

(**A**, **B**) The two most significant gene expression profiles were identified using Short Time-series Expression Miner. Profile 0 (5,411 genes) in (**A**) shows a continuous decrease, whereas Profile 1 (6,303 genes) in (**B**) shows an initial decrease followed by a later increase (both $p < 0.001$). (**C**) Cellular senescence at different OA stages was assessed using β-galactosidase staining. Black arrows at 1-week post-surgery (W1) indicate early positive senescence cells. The boxed area at 6 weeks post-surgery (W6) shows strong β-gal staining in the medial middle (MM) region, but no staining in the medial outer (MO) region due to cell death (blue arrow). Scale bars: 200 µm.

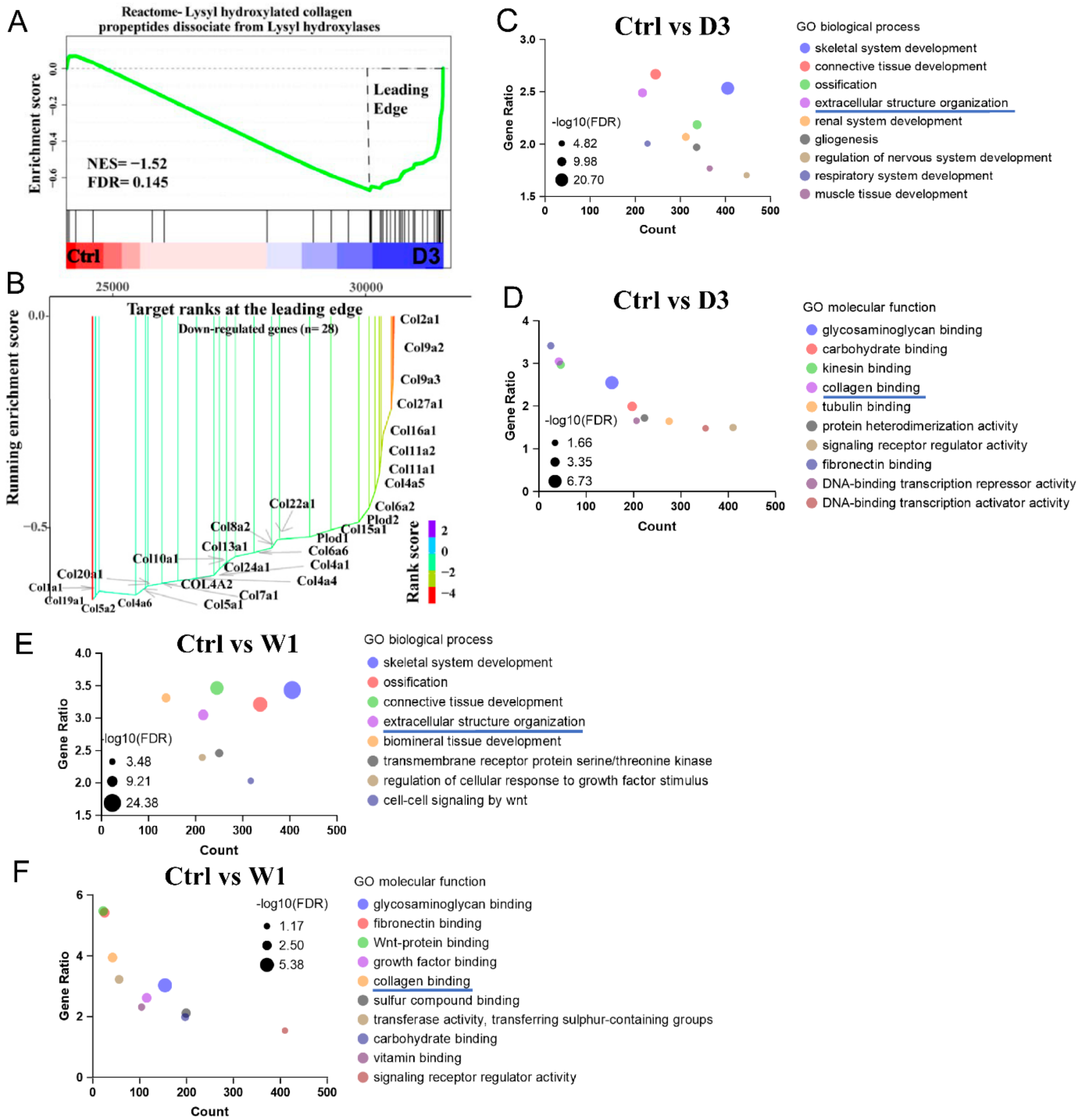


**Fig. S6. GSEA and GO enrichment pathways in pre-osteoarthritis (OA).**
(**A**) GSEA Reactome pathway analysis highlighting an enriched hallmark gene set (**B**) of lysyl hydroxylated collagen peptides dissociation, which is downregulated in this pathway at D3 compared with that in Ctrl. NES = -1.52, FDR = 0.145. (**C**, **D**) Bubble plots showing Gene Ontology (GO) enrichment analysis of differentially expressed genes (DEGs) during early OA stages. GO terms enriched at 3 days post-OA induction (D3) for biological processes (**C**) and molecular functions (**D**). (**E**, **F**) GO terms enriched at 1-week post-OA induction (W1) for biological processes (**E**) and molecular functions (**F**).

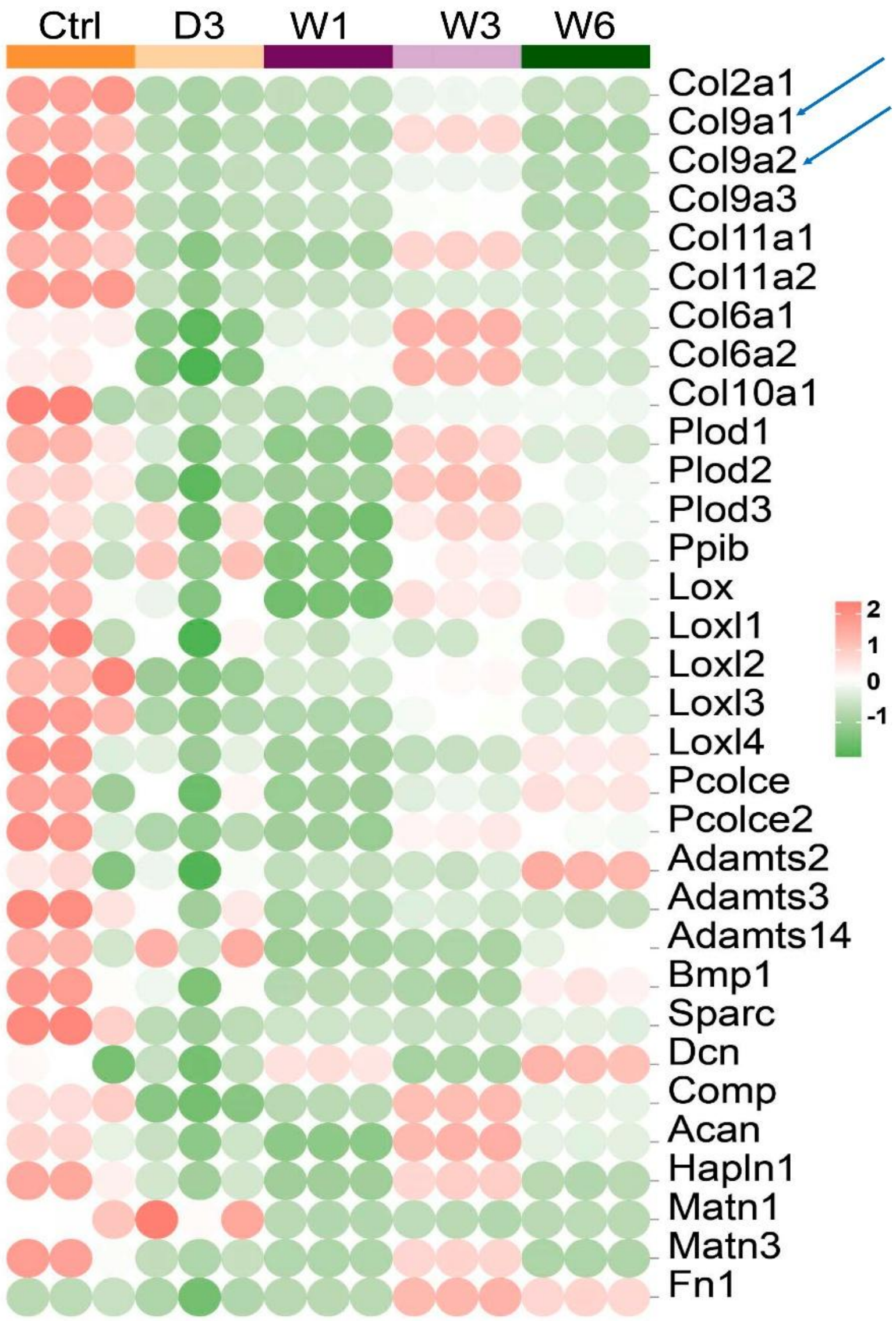


**Fig. S7. Heatmap of extracellular matrix (ECM)-related differentially expressed genes across cartilage samples at various osteoarthritis (OA) stages.**

Heatmap of ECM network–related gene expression in rat tibial cartilage at intact (Ctrl), day 3 (D3), and 1- (W1), 3- (W3), and 6-week (W6) post-surgery. Each row is a gene and each column a time point; color represents normalized expression. Multiple ECM transcripts, including *Col9a1* and *Col9a2*, are markedly downregulated at the pre-OA stage (D3 and W1).

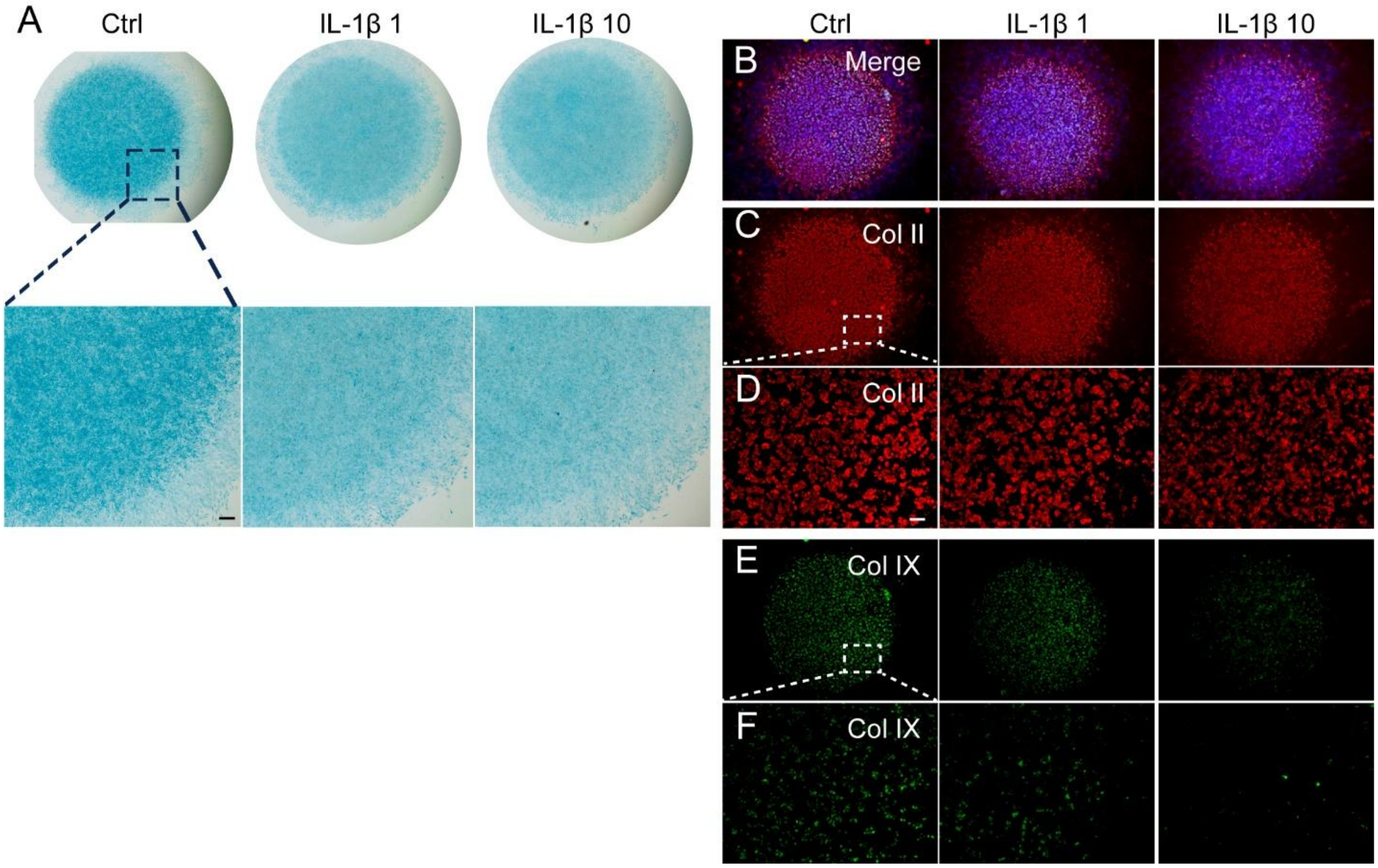


**Fig. S8. IL-1β-induced matrix degradation in chondrocyte pellets.**
(**A**) Alcian blue staining of chondrocyte pellets treated with increasing concentrations of IL-1β (1 or 10 ng/mL) reveals a dose-dependent loss of proteoglycans, indicating extracellular matrix degradation. Scale bar: 200 µm. Magnified regions from the dashed black boxes are shown below. (**B**–**F**) Immunofluorescence staining of chondrocyte pellets to assess collagen expression in response to IL-1β. (B) Merged images of pellets stained with DAPI (blue), collagen type II (Col II, red), and collagen type IX (Col IX, green). (**C**, **D**) Col II expression under control (Ctrl), IL-1β 1 (1 ng/mL), and IL-1β 10 (10 ng/mL) conditions, with (**D**) showing high-magnification views from the boxed regions in (**C**). (**E**, **F**) Col IX expression at the corresponding regions is shown in (**C**, **D**), with (**F**) presenting magnified regions from (**E**). Col IX expression markedly decreased with IL-1β treatment, whereas Col II was only modestly affected. Scale bars: 100 µm.

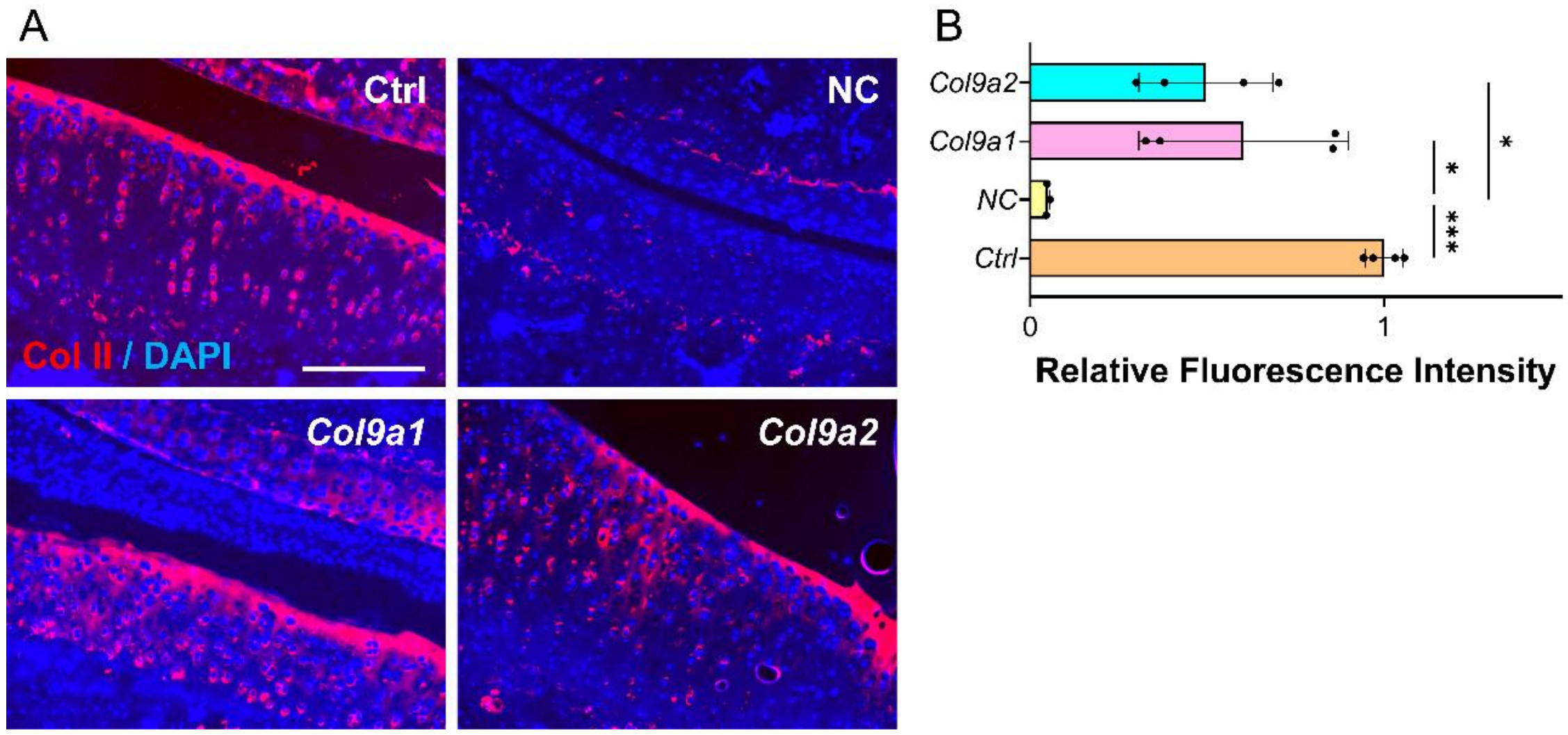


**Fig. S9.** ***Col9*** **mRNA treatment preserves Col II expression.**
(**A**) Representative immunofluorescence staining of Col II in cartilage sections after mRNA treatment, and (**B**) quantification of the corresponding fluorescence intensity. Scale bar: 200 µm. Statistical significance was determined using one-way ANOVA followed by Tukey's multiple comparisons test (*$p < 0.05$, ***$p < 0.001$).

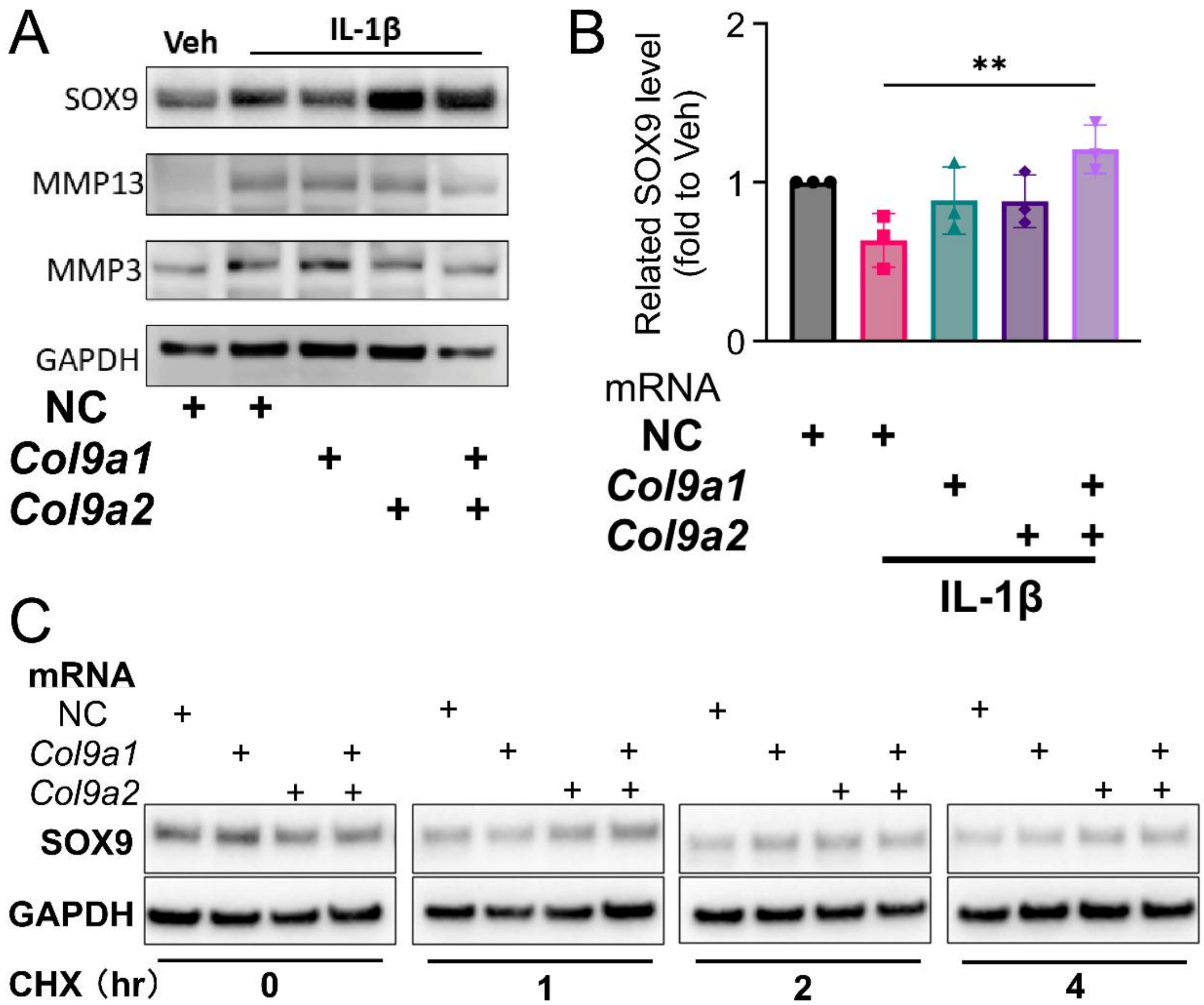


**Fig. S10.** ***Ex vivo*** **co-treatment with** ***Col9*** **mRNAs suppresses IL-1β–induced inflammation and preserves SOX9 expression.**

(**A**) Representative western blots showing the expression of key anabolic (SOX9) and catabolic (MMP13, MMP3) markers under each treatment condition. (**B**) Quantitative analysis of SOX9 protein levels from western blot data (see Materials and Methods for replicate structure). (**C**) Cycloheximide (CHX) chase assay showing reduced SOX9 degradation in mRNA-treated chondrocytes compared to the non-targeted control.

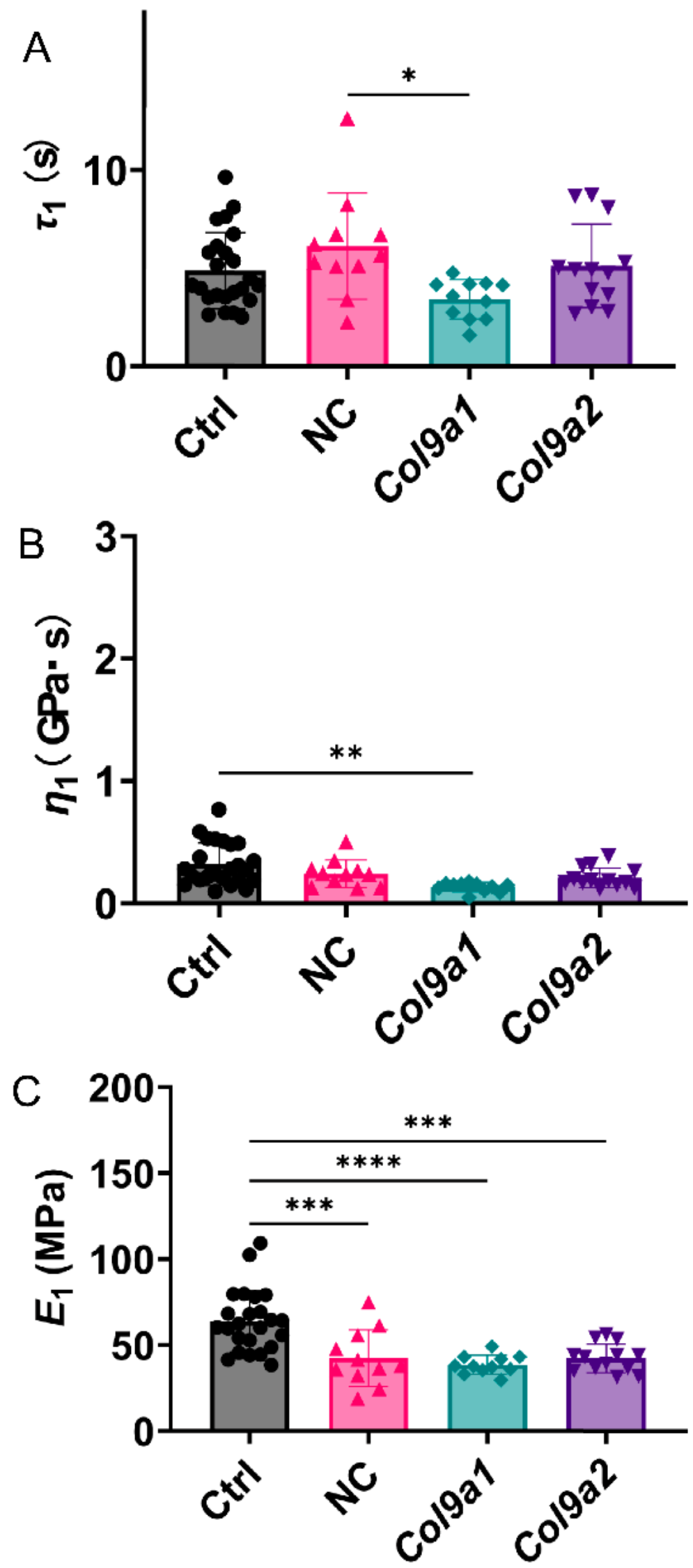


**Fig. S11. Viscoelastic parameters of pre-osteoarthritis (OA) cartilage following *Col9* mRNA administration.**

Parameters derived from the first element of the Kelvin–Voigt model, including (**A**) first retardation time ($\tau_1$), (**B**) first viscosity ($\eta_1$), and (**C**) first elastic modulus ($E_1$).

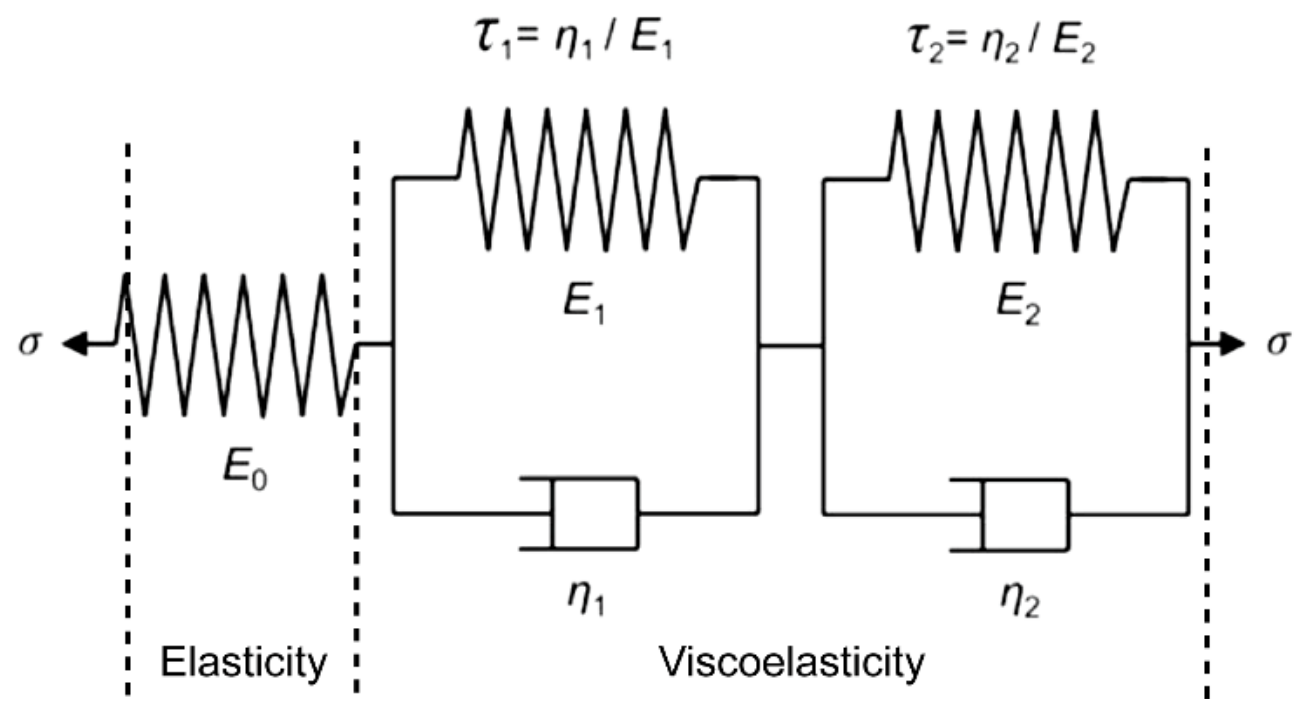


**Fig. S12. Schematic diagram of the generalized Kelvin–Voigt model.**
One elastic spring in series with two parallel spring-dashpot bodies, used to separate the elastic and viscoelastic responses of cartilage during creep.

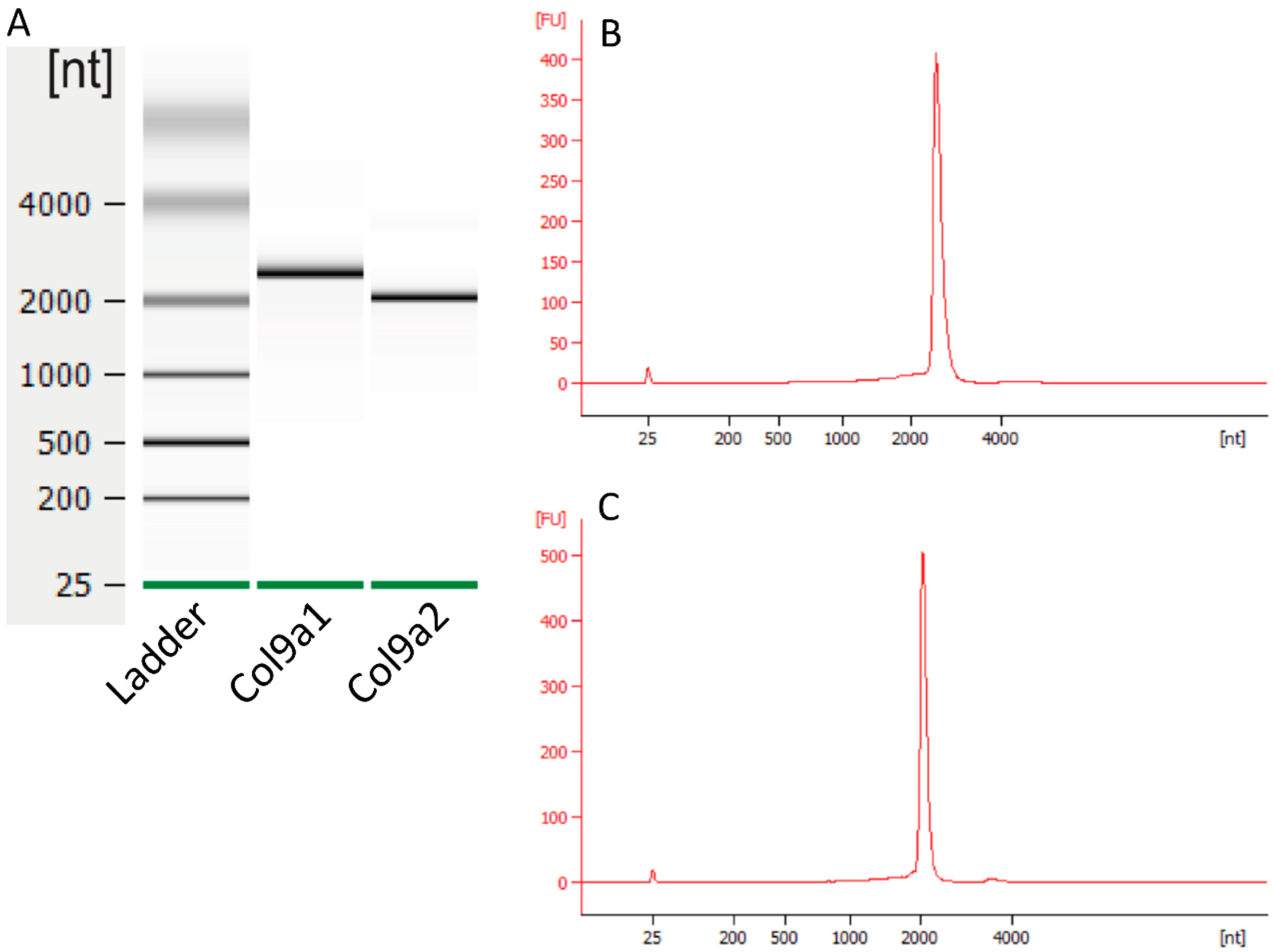


**Fig. S13. Bioanalyzer analysis of synthesized *Col9* mRNAs.**
(**A**) Gel image from the Agilent 2100 Bioanalyzer showing the size distribution of *in vitro* transcribed (IVT) mRNAs. Lane 1: RNA ladder; Lane 2: *Col9a1* mRNA; Lane 3: *Col9a2* mRNA. Major bands above 2000 nucleotides (nt) correspond to the expected transcript sizes. (**B**, **C**) Electropherograms corresponding to *Col9a1* and *Col9a2* mRNAs, respectively, showing single sharp peaks at their expected sizes, confirming high purity and integrity of the mRNA preparations.

| Gene name | | Sequence (5’-3’) |
| --- | --- | --- |
| *GAPDH* | Forward primer | GGGTGTGAACCACGAGAAAT |
| | Reverse primer | ACTGTGGTCATGAGCCCTTC |
| *Col9a1* | Forward primer | TATCCCAGTGGACTGCCTGA |
| | Reverse primer | CCCGTCCAGTCCCTTGTATG |
| *Col9a2* | Forward primer | GAAGCCTGGCATTGATGGTTT |
| | Reverse primer | ACAAGAAATCCGCACTCCCTT |
| *Mmp13* | Forward primer | CCCTGATGTTTCCCATCTATAC |
| | Reverse primer | GTCACACTTCTCTGGTGTTT |
| *Mmp3* | Forward primer | CAATCCCTCTATGGACCTCCC |
| | Reverse primer | CCCTCCATGAAAAGACTCAGAGG |
| *Sox9* | Forward primer | AAGACCACCCCGATTACAAGT |
| | Reverse primer | CCCCTCTCGTTTCAGATCAACT |

**Table S1. Primer sequences for RT-qPCR.**

Forward and reverse primer sequences for target genes (*Col9a1, Col9a2, Mmp13, Mmp3, Sox9*) and the reference gene *GAPDH*.
